# Contributions of historical and contemporary geographic and environmental factors to phylogeographic structure in a Tertiary relict species, *Emmenopterys henryi* (Rubiaceae)

**DOI:** 10.1038/srep24041

**Published:** 2016-05-03

**Authors:** Yong-Hua Zhang, Ian J. Wang, Hans Peter Comes, Hua Peng, Ying-Xiong Qiu

**Affiliations:** 1 Key Laboratory of Conservation Biology for Endangered Wildlife of the Ministry of Education, and College of Life Sciences, Zhejiang University, Hangzhou 310058, China; 2 Department of Environmental Science, Policy, and Management, University of California, Berkeley, CA 94720, USA; 3 Department of Ecology & Evolution, Salzburg University, A-5020 Salzburg, Austria; 4 Laboratory of Biodiversity and Biogeography, Kunming Institute of Botany, Chinese Academy of Sciences, Kunming, Yunnan 650204, China

## Abstract

Examining how historical and contemporary geographic and environmental factors contribute to genetic divergence at different evolutionary scales is a central yet largely unexplored question in ecology and evolution. Here, we examine this key question by investigating how environmental and geographic factors across different epochs have driven genetic divergence at deeper (phylogeographic) and shallower (landscape genetic) evolutionary scales in the Chinese Tertiary relict tree *Emmenopterys henryi*. We found that geography played a predominant role at all levels – phylogeographic clades are broadly geographically structured, the deepest levels of divergence are associated with major geological or pre-Quaternary climatic events, and isolation by distance (IBD) primarily explained population genetic structure. However, environmental factors are clearly also important – climatic fluctuations since the Last Interglacial (LIG) have likely contributed to phylogeographic structure, and the population genetic structure (in our AFLP dataset) was partly explained by isolation by environment (IBE), which may have resulted from natural selection in environments with divergent climates. Thus, historical and contemporary geography and historical and contemporary environments have all shaped patterns of genetic structure in *E. henryi*, and, in fact, changes in the landscape through time have also been critical factors.

Understanding the contemporary and historical ecological (climatic, geographical) factors shaping population genetic diversity, structure, and divergence is of great interest to molecular ecology, evolutionary biology and conservation biology^1^,^2^,^3^. Populations separated by physical geographical barriers (including geographic distance) may diverge under any combination of natural selection and random genetic drift resulting from reduced gene flow and population connectivity^4^,^5^. In the absence of extrinsic (e.g. physical) barriers to gene flow, population divergence may still occur when reproductive isolation evolves between populations as a result of ecologically-based divergent selection in different environments^5^,^6^,^7^,^8^,^9^,^10^. Thus, population genetic divergence can result from both geographical and environmental factors (including climate and soil, among others). Disentangling the roles of geographic and environmental forces in driving genetic structure during certain periods (usually contemporary) has seen a large body of research in both plants and animals over recent years^4^,^11^,^12^,^13^,^14^,^15^. However, few studies have examined how these factors contribute to genetic structure through time, including both historical and contemporary geographic and environmental factors, to better understand how changing climates and geographic landscape features can influence patterns of genetic structure observed presently^16^,^17^.

For example, climatic fluctuations during the Quaternary which resulted in population isolation in multiple refugia are considered major drivers of population divergence and broad phylogeographic patterns^18^,^19^,^20^. Of course, major geographic barriers, like oceans, rivers, and mountains, are also recognized as key drivers of biogeo-graphic structure^18^, and thus, both environmental and geographic factors can contribute to genetic divergence at deeper evolutionary scales. Likewise, geography and the environment have also been recognized as critical factors underlying genetic differentiation over shorter evolutionary scales, like the evolution of population genetic structure among contemporary populations on a landscape^5^,^21^,^22^.

So, how do historical and contemporary geographic and environmental factors contribute to genetic divergence at different evolutionary scales? This is a central yet largely unexplored question in ecology and evolution^16^,^17^. In general, the flora of subtropical (Central-Southeast) China presents some excellent systems for such studies, including several genera of Tertiary relict trees that have inhabited topographically and ecologically heterogeneous environments (in terms of climate, soil, etc.) for millions of years^23^,^24^,^25^,^26^. These genera (e.g. *Cathaya*, *Ginkgo*, *Metasequoia*, *Davidia*, *Emmenopterys*) are thought to represent remnants of the so-called ‘boreotropical flora’ that likely formed a belt of vegetation around the Northern Hemisphere during the Early Tertiary/Eocene^27^,^28^,^29^. Here, we examine this key question by investigating how environmental and geographic factors across different epochs have driven genetic divergence at deeper (phylogeographic) and shallower (landscape genetic) evolutionary scales in the Chinese flowering tree *Emmenopterys henryi* Oliv. (Rubiaceae).

*Emmenopterys henryi* is a particular suitable species for addressing these issues. This deciduous tree, which is the only extant species of its genus, is native to subtropical China, where it occurs in disjunct montane valleys of mainly warm-temperate deciduous (WTD) forests, at elevations ranging from *c*. 400–1600 (2000) m above sea level^30^ (Fig. 1A). Landscape characteristics, climatic conditions, and soil types vary between regions within the distribution range of *E. henryi*^31^. Well-preserved infructescences of now-extinct *Emmenopterys* species are known from the Eocene of North America and Germany^32^, but there are no reliable fossils of *E. henryi*. However, there is dated molecular evidence to suggest that the origin of this species dates back to the Early Miocene^33^. Previous results based on inter-simple sequence repeat (ISSR) markers indicate that *E. henryi* exhibits significant population genetic structure and divergence^34^; however it remains unclear whether this is the result of long-term geographical barriers to gene flow, ecologically-based divergent selection, or recent habitat fragmentation.

In this study, we integrate genetic markers that capture signatures from historical (chloroplast DNA) and contemporary (amplified fragment length polymorphisms; AFLPs) divergence^35^, ecological niche modelling (ENM), and spatial genetic modelling approaches to disentangle the relative roles of geography, climate, and ecology in shaping the population genetic structure of *E. henryi* across subtropical China. Our main objectives were to: (i) estimate the timing and pattern of divergence among populations of *E. henryi*; (ii) investigate how climatic and geographical variation over space and time explain patterns of phylogeographic and population genetic structure; and (iii) explore the specific environmental variables that may underlie local adaptation through natural selection in divergent environments.

## Results

### cpDNA and ITS phylogeography and diversity

**Figure 1:**
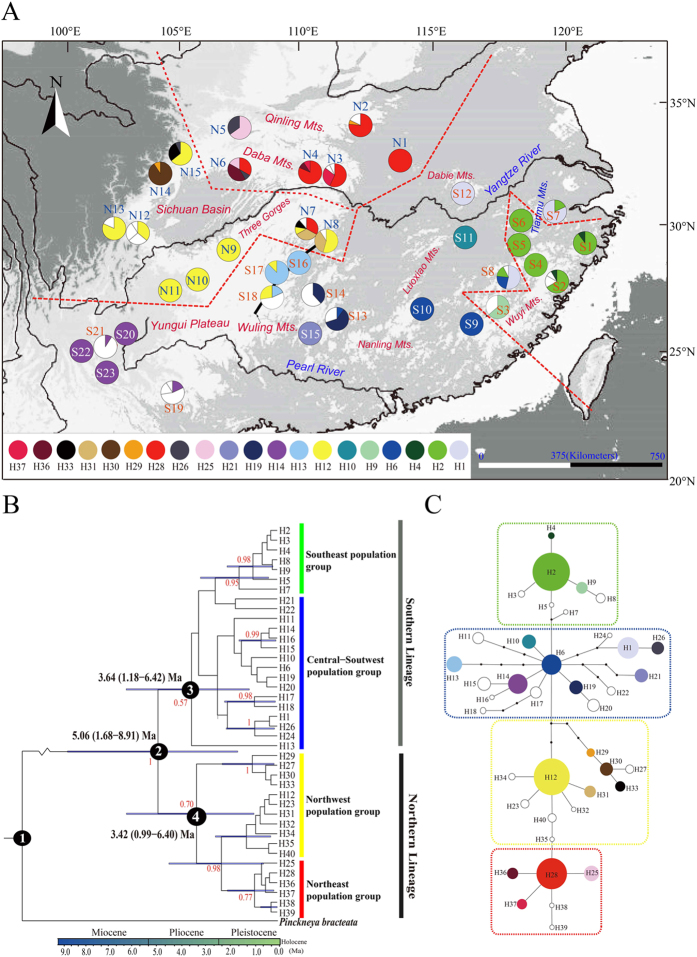
Analysis of cpDNA (*psb*A*–trn*H, *trn*L*–trn*F, *trn*T*–trn*L) haplotypes of *Emmenopterys henryi*. **(A)** Geographical distribution of haplotypes across 38 sampled populations. Pie charts represent haplotype proportions. Colored haplotypes are shared by two or more populations, and blank ones are private haplotypes. Population groups identified by SAMOVA for *K* = 4 are delimited by red dotted lines. **(B)** Fifty percent majority-rule consensus tree of the Bayesian phylogenetic analysis. Numbers on branches are Bayesian posterior probabilities. **(C)** Statistical parsimony network of cpDNA haplotypes. Lines represent single nucleotide substitutions, and the small black dots indicate missing haplotypes (extinct or not found). The sizes of circles are approximately proportional to sample size (*n*), with the smallest circles representing *n* = 1 and the largest representing *n* = 64. The map was drawn using ArcGIS v.9.3 (ESRI, Redlands, CA, USA)^94^.

The three cpDNA-IGS regions surveyed across the 443 individuals of *E. henryi* were aligned along a total length of 2163 bp with 26 single-site mutations (including two 1-bp indels), 18 length polymorphisms (2–78 bp) and one inversion (27 bp) observed (Table S5). A total of 40 haplotypes (‘chlorotypes’; H1–40) were detected in the 38 *E. henryi* populations across subtropical China (Table S1; Fig. 1A). Of the 40 haplotypes, 20 were shared by at least two populations while the other 20 haplotypes were only found in a single population (Table S1; Fig. 1A). The most common haplotypes were H12 (found in 10 populations with a frequency of 0.25), H2 (17.5% of all populations), H28 (15%), H14 (12.5%), and H6 (10%). Total haplotype diversity (*h*_T_) was estimated to be 0.928 and within-population diversity (*h*_S_) was 0.332 (Table S1). Regression analyses showed that *h*_S_ was not dependent on longitude or latitude (*P* = 0.23, 0.66, respectively), but *π*_s_ was weakly related to latitude (*R*^*2*^ = 0.116, *P* = 0.036).

The Bayesian haplotype tree from beast supported the monophyly of *E. henryi* [posterior probability (*PP*) = 1], and two main (‘Northern’ vs. ‘Southern’) lineages were recognized with weak support (*PP* = 0.57, 0.70, respectively; Fig. 1B). The haplotypes of the Northern lineage were located in the northern part of the species’ range, except for H12 from populations S17 and S18, while those of the Southern lineage were present only in southern populations (Fig. 1A). In the haplotype network, the two major lineages are also recognized (Fig. 1B,C). Most haplotypes close to each other in the phylogeny and haplotype network tended to occur in nearby populations (Fig. 1). In the samova analysis, *F*_CT_ values increased progressively as the value of *K* increased from 2 to 26. However, with *K* ranging between 5 and 26, *F*_CT_ values did not increase significantly, and in most cases the newly defined groups comprised single populations (Fig. S1). Thus, we retained the configuration of *K* = 4 (*F*_CT_ = 0.523). The four cpDNA groups identified are populations S1–S6 in the east (Southeast group), S7–S23 in the middle (Central-Southwest group), N1–N6 in the northeast (Northeast group), and N7–N15 in the northwest (Northwest group). This grouping is mostly consistent with the phylogenetic analyses (Fig. 1B,C).

Non-hierarchical AMOVA (Table 1) revealed a strong population genetic structure for cpDNA sequence variation at the species level (*Φ*_ST_ = 0.779; *P *< 0.001). Hierarchical AMOVA, however, revealed that of the total genetic variation, 36.48% was distributed between the Northern lineage and the Southern lineage (*Φ*_CT_ = 0.365), 45.43% was explained by variation among populations within regions (*Φ*_SC_ = 0.715), and only 18.10% was found within populations (*Φ*_ST_ = 0.819; Table 1). Nevertheless, there was greater population subdivision in the Southern lineage (*Φ*_ST_ = 0.823) when compared to the Northern lineage (*Φ*_ST_ = 0.551; Table 1). Moreover, significant phylogeographic structure for cpDNA was observed at the range-wide scale (*G*_ST_/*N*_ST_ = 0.704/0.802, *P *< 0.01) and in the Southern lineage (*G*_ST_/*N*_ST_ = 0.757/0.868, *P *< 0.05), but no such structure was found in the Northern lineage (*G*_ST_/*N*_ST_ = 0.521/0.379, *P* > 0.05; Table 1). Finally, Mantel tests uncovered a strong pattern of IBD for cpDNA both at the range-wide scale (*r* = 0.237, *P* = 0.001) and the region scale (Northern lineage: *r* = 0.506, *P* = 0.001; Southern lineage: *r* = 0.293, *P* = 0.001).

**Figure 2:**
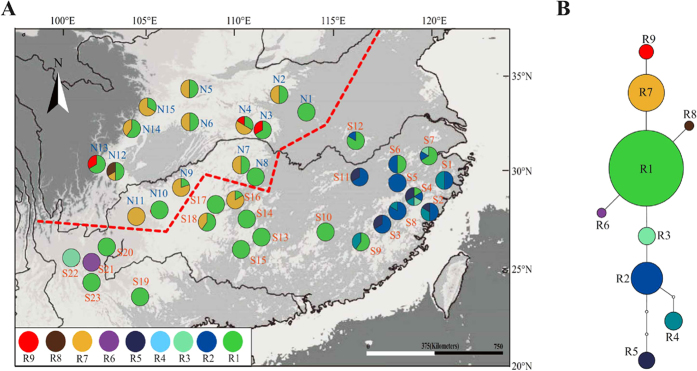
Analysis of internal transcribed spacer (ITS) ribotypes of *E. henryi*. **(A)** Geographical distribution of ITS ribotypes across sampled populations. Pie charts represent ribotype proportions. **(B)** Statistical parsimony network of ITS ribotypes. Lines represent single nucleotide substitutions, and the small white dots indicate missing ribotypes (extinct or not found). The sizes of circles are approximately proportional to sample size (*n*), with the smallest circles representing *n* = 2 and the largest representing *n* = 110. The map was drawn using ArcGIS v.9.3 (ESRI, Redlands, CA, USA)^94^.

The ITS sequences of 212 individuals (38 populations) of *E. henryi* were aligned with a total of length of 767 bp, exhibiting 10 nucleotide substitutions (ITS-1: 4; ITS-2: 6; Table S6). Together, these 10 polymorphic sites identified nine ITS haplotypes (‘ribotypes’, R1–9; Table S6). Of those, five ribotypes were specific to the Southern cpDNA lineage (R2–6) and three to the Northern cpDNA lineage (R7–9; Fig. 2A). These lineage-specific ribotypes formed separate tcs clades, except for the shared ribotype R1 (Fig. 2B).

### Molecular dating and historical demography based on cpDNA sequence variation

Based on the assumption that *E. henryi* and *P. bracteata* diverged at *c*. 22 Ma (Manns *et al*.^33^; Fig. 1B; node 1), the time since divergence between the Northern and Southern lineages was estimated by beast analysis to have occurred 5.06 Ma (95% HPD: 1.68–8.91 Ma; node 2). The onset of lineage diversification was estimated as 3.42 Ma (Northern lineage, 95% HPD: 0.99–6.40 Ma; node 4) and 3.64 Ma (Southern lineage, 95% HPD: 1.18–6.42 Ma; node 3). For this cpDNA-IGS chronogram, we recovered an average substitution rate of 6.225 × 10^−10^ s/s/y by the beast analysis, which is much lower than the average values generally reported for noncoding regions of the chloroplast genome (e.g. 1.2–1.7 × 10^−9^ s/s/y)^36^; but in accordance with that in woody taxa and⁄or phylogenetic relicts (viz. ‘living fossils’)^37^,^38^,^39^,^40^.

For the four cpDNA population groups of *E. henryi*, at least one of the estimates of Tajima’s *D* and Fu’s *F*_S_ was generally significant, except for the Central-Southwest group (Table 2). In contrast, almost all four groups failed to reject the spatial expansion model (*SSD*, *H*_Rag_ values *P* > 0.05), with the exception of the Northeast group (*P *< 0.05; Table 2). In addition, the mismatch distributions of the Southeast, Northeast and Northwest groups were unimodal, except for the Central-Southwest group (Fig. S3). Consequently, the Southeast, Northeast and Northwest groups were found to fit to the distributions expected under a spatial expansion model. Based on the corresponding *τ* values, and assuming a substitution rate of 6.225 × 10^−10^ s/s/y (see above), we dated the three spatial expansions to the last glacial cycle(s) (Southeast: *c*. 0.23 Ma, 95% CI: 0.000–0.811 Ma; Northwest: *c*. 0.19 Ma, 95% CI: 0.111–0.314 Ma; Northeast: *c*. 0.26 Ma, 95% CI: 0.133–0.376 Ma; Table 2).

### AFLP analysis

The nine primer combinations employed with samples from 37 populations (394 individuals) of *E. henryi* generated a total of 457 fragments, of which 431 (94.31%) were polymorphic. Different primer pairs amplified variable numbers of fragments, from 38 to 69, with an average of 50.8 ± 10.1 fragments per primer combination (Table S3). The percentage of polymorphic fragments varied among primer pairs from 88.89 to 98.55% (Table S3). Because of the high degree of polymorphism, these primer combinations distinguished all 394 individuals as separate phenotypes. AFLP variation within populations (in terms of *PPF*, *I*, *H*_E_, *DW*) varied widely (Table S1). The highest genetic diversity was observed in a northern population (N12), with *PPF* = 74.18%, *I* = 0.243, and *H*_E_ = 0.162. By contrast, genetic diversity was lowest in a southern population (S22), with *PPF* = 57.77%, *I* = 0.035, and *H*_E_ = 0.023. Regression analyses showed that *H*_E_ was weakly related to latitude (*R*^2^ = 0.110, *P* = 0.045).

**Figure 3:**
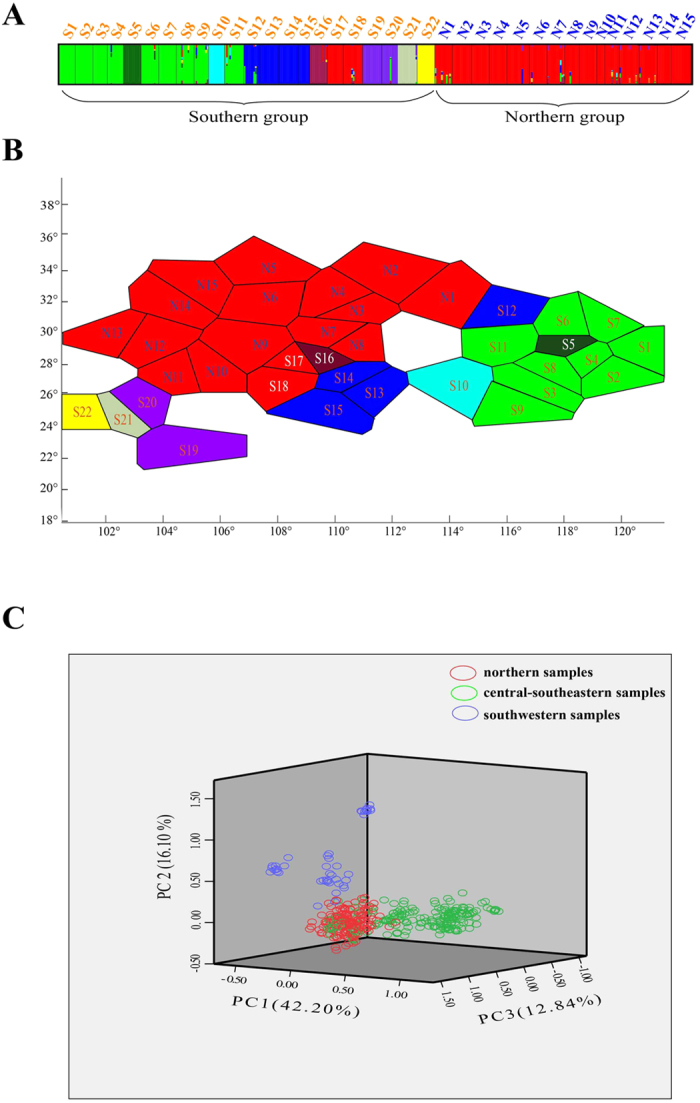
Results for 37 populations of *E. henryi* based on the analysis of 457 AFLP loci. **(A)** Histogram of the baps assignment test for 37 populations (394 individuals) (*K* = 9). Each vertical bar represents an individual. Southern and northern cpDNA lineages contain 9 and 1 AFLP clusters, respectively. Population codes are shown above the bar. **(B)** Spatial clustering of 37 populations in baps (*K* = 9). Each polygon represents one population corresponding to the histogram above, and colours of polygons indicate clusters. **(C)** PCoA for 37 populations (394 individuals). Percentages of total variance explained by the first three coordinates are shown on the respective axes.

The partition with the highest log marginal likelihood (spatial: -51313.01, non-spatial: -51225.49) produced by baps identified nine different clusters (Fig. S2A). All populations from the Northern and two (S17, S18) from the Southern (cpDNA) lineage formed one cluster, while the remaining populations from the Southern lineage were further divided into eight clusters (Fig. 3A,B). In the PCoA-plot, the first axis (42.20% of the variation) and the third axis (12.84%) indicated that the main split in the dataset was between the central-southeastern and the northern samples, while the second axis (16.10%) separated the southwestern samples from the northern group (Fig. 3C). The NJ analysis based on genetic distances among populations also identified three regional groups, again comprising populations from the central-southeastern region (67% bootstrap support), southwestern region (92%), and northern region (<50%; Fig. S2B). With few exceptions, S16 and S17 clustered with the northern group. Populations from central-southeastern and southwestern China showed higher among-population divergence when compared with those from within the northern group (Figs 3C and S2B). Non-hierarchical AMOVA indicated high overall levels of population differentiation for AFLPs in *E. henryi* (*Φ*_ST_ = 0.344; Table 1). When accounting for the species’ significant hierarchical (regional) substructure (*Φ*_CT_ = 0.061), levels of population subdivision still remained high (*Φ*_SC_ = 0.364), but were markedly higher in the Southern lineage (*Φ*_ST_ = 0.416) than in the Northern one (*Φ*_ST_ = 0.175; Table 1).

### Ecological niche modelling across temporal scales

**Figure 4:**
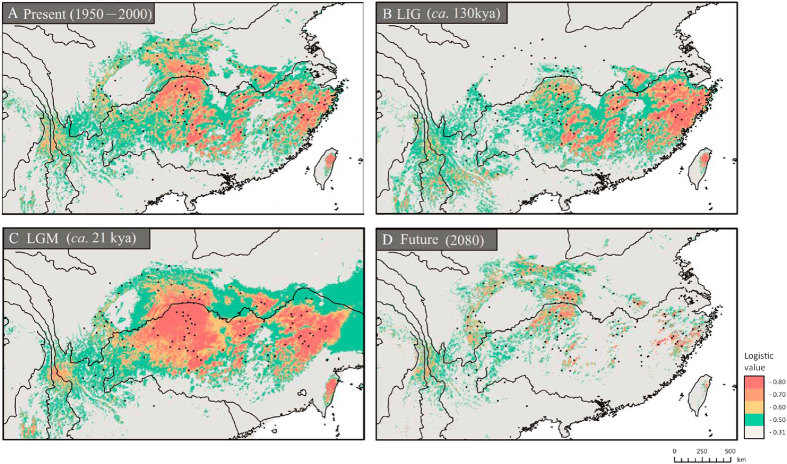
Potential distributions as probability of occurrence for *E. henryi* in China **(A)** at present (1950*–*2000), **(B)** at the Last Interglacial (LIG; *c*. 130kya), **(C)** at the Last Glacial Maximum (LGM; *c*. 21kya), and **(D)** in the future (2080, A2 scenario). Ecological niche models were established with current bioclimatic variables on the basis of extant occurrence points (black dots) of the species using maxent v.3.2.1. Predicted distribution probabilities (in logistic values) are shown in each 2.5 arc-min pixel. Maps were generated using ArcGIS v.9.3 (ESRI, Redlands, CA, USA)^94^.

The maxent model for *E. henryi* had high predictive power and did not overfit the presence data (AUC = 0.804 ± 0.003). The current distributional predictions (Fig. 4A) were accurate representations of the species’ extant distribution, except for some predicted areas where the species does not occur at present (e.g. southeastern Qinghai–Tibetan Plateau and Taiwan). Palaeodistribution modeling suggested more restricted ranges of the species during the Last Interglacial (LIG) compared with its current distribution, particularly in north-central China (e.g. northern Sichuan Basin and Daba/Qinling Mts.; Fig. 4B), with subsequent expansion at the Last Glacial Maximum (LGM) to cover a slightly greater area than that predicted under current climatic conditions (Fig. 4A,C). However, ENMs for the future (2080) predict that climate change will result in a reduction of the species’ potential range in China. Most evident is a loss of suitable habitat in areas south of the Yangtze Delta, where only small and disjunct mountain areas are predicted as suitable (Fig. 4D).

### IBD and IBE

The structural equation modelling (SEM) and multiple matrix regression with randomization (MMRR) analyses both revealed significant effects of IBD and IBE on neutral (non-outlier) AFLP divergence (*F*_ST_) in this species. In both analyses, IBD explained more of the variation in genetic differentiation than IBE (SEM: ≈2 times as much; MMRR: ≈1.25 times as much; Table 3). SEM identified significant pairwise relationships among environmental distance, geographic distance, and genetic distance (*P *< 0.01) and estimated that IBD (β_D_ = 0.360 ± 0.037) contributed about twice as much as IBE (β_E_ = 0.181 ± 0.151) to genetic distance. Similarly, MMRR also estimated the contribution of IBD (β_D_ = 0.307, *P *< 0.001) to be larger than that of IBE (β_E_ = 0.247, *P* = 0.002). Each analysis detected low levels of covariation between geographical and environmental distance (0.133 ± 0.028 in SEM and 0.488 in MMRR), although the covariation based on the MMRR analysis was approaching the reliability limit of 0.5 (Table 3). The difference between these estimates from each analysis is due to the different environmental distance matrices used, estimated as Euclidian distances among all observed environmental variables in MMRR and as a latent variable constructed from the observed environmental variables in SEM.

For the SEM analysis, we also quantified the contributions of the individual environmental variables to the composition of the environmental dissimilarity latent variable (Table S7). The results revealed that bio4 (temperature seasonality) and bio7 (temperature annual range) were the primary contributors, followed by bio1 (annual mean temperature), bio5 (max temperature of warmest month), bio9 (mean temperature of driest quarter), and bio15 (precipitation seasonality). By contrast, slope, soil, and bio12 (annual precipitation) had very minor contributions.

### Detecting potential loci under selection: outlier loci test and MLR analysis

**Figure 5:**
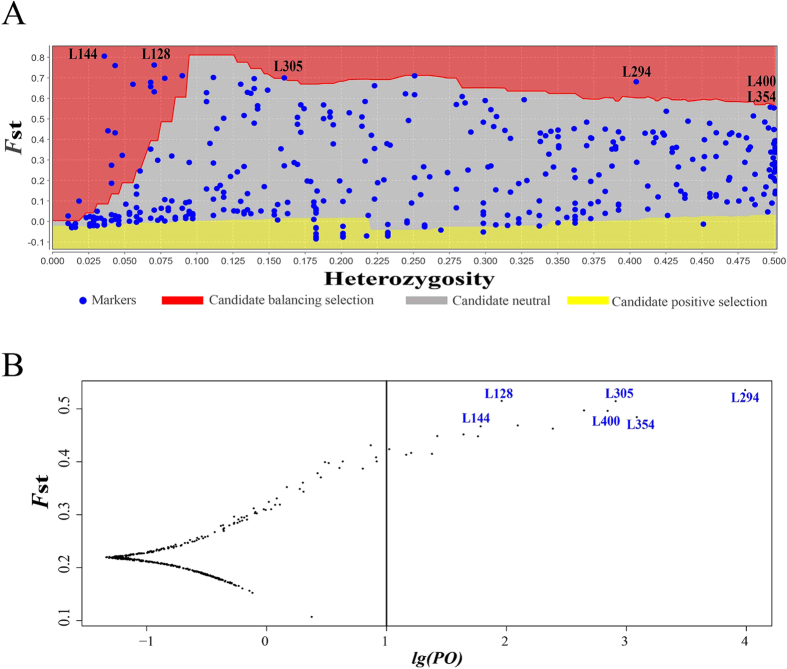
The outlier tests for *E. henryi* using multi-methods based on 457 AFLP loci. The outlier loci were detected by fdist in mcheza **(A)** and by bayescan **(B)**, respectively, among the nine genetic clusters identified from the baps analysis. Six outlier loci were identified by both methods together.

Using fdist, 67 of 457 loci (14.66%) showed high probability (99.5%) for divergent selection among the nine baps groups, including 21 loci under directional selection and 46 loci under balancing selection (Fig. 5A). Additionally, we identified 16 outlier loci in bayescan (3.50% of all 457 loci; Fig. 5B). However, only six loci were identified by both fdist and bayescan (Fig. 5). Eventually, four potential loci under selection (L128, L144, L294, and L305) were confirmed by the MLR analysis with 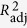 > 0.5 (Table S8). When we ran linear regressions using each environmental variable individually, all six loci were significantly (*P *< 0.05) associated with at least two of the eight selected environmental variables (Table S7). Among the environmental variables, bio2 (mean diurnal temperature range), bio4 (temperature seasonality), bio5 (maximum temperature of the warmest month), bio12 (annual precipitation) and bio15 (precipitation seasonality) were most highly associated with the potential loci under selection.

## Discussion

The results of our phylogeographic and landscape genetic analyses reveal how historical and contemporary environmental and geographic factors have all contributed to presently observed patterns of genetic divergence in *E. henryi*. At broad scales, the results of our cpDNA and ITS phylogeography show that genetic variation is, largely, geographically structured. In the cpDNA phylogeography, we found two major lineages corresponding to the Northern and Southern populations (with minor exceptions). The boundary between these lineages is located in the region of the Yangtze River and next to the Three Gorges Mountain Region (TGMR; Figs 1, 2, 3). We found evidence of admixture of Northern lineage cpDNA haplotypes into the Southern lineage (S16, S17, S18), which is most likely explained by both introgression following secondary contact during glacial periods and incomplete lineage sorting due to recent, postglacial divergence. Nuclear ITS ribotype data did not show such a distinct division between the Northern and Southern populations but did, nevertheless, indicate geographically structured ribotype frequency differences between these sets of populations and the presence of ribotypes found only in the Northern and Southern lineages. In fact, of the nine ribotypes we detected, only one was shared between lineages (R1), which was found in many populations, including those around the Yungui Plateau (Fig. 2). Several ribotypes and chlorotypes are also unique to Southeastern populations, where the climate is warmest and wettest, or to the Northernmost populations, where the climate is coolest and driest. Whether these haplotypes and populations sharing similar haplotype frequencies are clustered in these areas because they are environmentally divergent from the intermediate climate of central China, where the most haplotype diversity is found, or because of geographical restrictions to the mountain chains in these regions is still unclear. Nevertheless, it appears that major geographic regions harbor phylogeographic structure, but that geographic distances and major geographic barriers (like the Yangtze River) are not always major barriers to genetic introgression. For example, there is cpDNA evidence to suggest that two populations from the Northern lineage (N6 and N15 in the Qinling Mts.) were shaped by dispersal events from a southern population (S12 in central China), as a chlorotype (H1) unique to this latter population differs from its derived haplotype (H26) in the Qinling Mts. by only one step (Fig. 1A,C; Table S1). These populations are separated by several hundred kilometers, suggesting that very long distance dispersal may be possible in this species, that these populations have some shared ancestry, or that they may have been more widespread and come in contact in the past, possibly during the LGM when there was much more suitable habitat available to *E. henryi* (Fig. 4). Hence, the observed phylogeographic patterns may also reflect historical factors, particularly relating to the expansion of suitable habitat during the LGM from heavily fragmented habitat during the LIG, followed by the restriction, again, of suitable habitat leading to the present disjunct montane distribution of *E. henryi* populations (Fig. 4).

Our ENM analysis through time suggested that *E. henryi* populations likely experienced cycles of expansion and retraction into and out of local refugia, and Quaternary climatic fluctuations may have played a role in generating phylogeographic structure as well^26^,^41^,^42^. In fact, the earliest diversification events in *E. henryi* are associated with major geological and climatic events. The estimated divergence time between the two major lineages (Northern and Southern) of *E. henryi*, at approximately the Mio-/Pliocene boundary [*c*. 5.06 Ma (1.68–8.91) Ma; see node 2 in Fig. 1B], coincides with global climate cooling during the Late Miocene/Early Pliocene^43^,^44^. This cooling is hypothesized to have been a key trigger of aridification in East Asia along with stronger winter and/or weaker summer monsoon circulations^45^,^46^. Concurrently, the mid-Pliocene abrupt uplift of the eastern Tibetan Plateau (*c*. 3.4 Ma)^46^,^47^ induced dramatic geomorphological changes in Southwest China. These climatic and geological changes may have triggered early lineage diversification in *E. henryi* through habitat fragmentation and the formation of physical barriers to gene flow^46^, which we see reflected in early branching events within each of the major Northern and Southern lineages beginning around 3.64 to 3.42 Ma (see nodes 3 and 4 in Fig. 1B). Such tectonic/climate-induced vicariance has also been invoked to explain similar patterns of north-south differentiation in other forest tree species from subtropical China (e.g. *Taxus wallichiana*^48^, *Cercidiphyllum japonicum*^39^, *Kalopanax septemlobus*^49^). Expansion of potential suitable habitat of *E. henryi* during the last glaciations, indeed, is supported by our ENM analysis, which indicates larger distribution ranges of this lineage at the LGM compared to both the LIG and present (Fig. 4A–C).

In addition to the interplay between geographic and climatic factors, population demography and colonization history also likely contributed to the observed pattern of phylogeographic structure. For instance, our phylogeographic and ENM analyses are consistent with the relatively recent expansion of *E. henryi* into the northernmost regions (Northwest and Northeast groups) of its range. These groups (particular the Northeast group; N1–6) harbor lower genetic diversity (Fig. S3) and the most derived haplotypes found in our *E. henryi* phylogeny (Fig. 1B) and tcs networks (Fig. 1C), which both suggest populations founded following range expansion. Additionally, the MDA indicated a spatial (and demographic) expansion within the Northwest and Northeast groups at *c*. 0.19 Ma (95% CI: 0.111–0.314 Ma) and *c*. 0.26 Ma (95% CI: 0.133–0.376 Ma; Table 2), respectively, possibly coinciding with the penultimate (Riss) glacial (*c*. 0.12–0.35 Ma), although, due to the broad confidence intervals, this interpretation requires some caution. In fact, the species is not predicted to have occurred in the northern Sichuan Basin and the Daba/Qinling Mts. during the LIG (Fig. 4B), i.e. when temperatures were at least 5 °C higher than at present^47^. Accordingly, we hypothesize that the climate might have warmed enough during the LIG to extirpate *E. henryi* from the above regions, followed by recolonization via northward expansion during the LGM (Fig. 4C). Notably, such a significant impact of the LIG has also been detected for other plants and animals in subtropical China (e.g. *Pinus kwangtungensis*^50^, *Aegithalos concinnus*^51^, *Parus monticolus*^52^).

The Southeast group may also carry signatures of expansion – these populations also have reduced haplotype diversity (Fig. S3A) and significantly negative Tajima’s *D* and Fu’s *F*_S_ statistics (Table 2). This provides strong evidence for a relatively recent spatial (and demographic) expansion in the Tianmu and Wuyi Mts. located southwest of the Yangtze Delta Region (YDR). Although the range of our time estimates for this expansion, again, is broad (approx. 0–0.81 Ma; Table 2), the respective point estimate (*c*. 0.23 Ma) once more coincides with the penultimate (Riss) glacial (see above). In addition to the ENM and MDA analyses (Figs 4B,C and S3A), this expansion scenario is further supported by vegetation reconstructions based on fossil pollen data, which indicate that the YDR still sustained patches of northern peripheral WTD forest in the lowland areas of East China during the LGM^53^. Thus, we again find evidence that Quaternary climatic oscillations across geographic regions of China likely contributed to phylogeographic patterns in this species.

Clearly, at broad phylogeographic scales, our results demonstrate distinct geographic genetic structure across the range of *E. henryi*. At finer spatial and evolutionary scales, our landscape genetic analysis of AFLP genotype data also revealed that geographical isolation played the predominant role in driving genetic divergence of *E. henryi* populations. However, we also found that environmental/climatic variation also contributed significantly to explaining population genetic structure (SEM: IBD ≈ 36.0 vs. IBE ≈ 18.1%; MMRR: IBD ≈ 30.72 vs. IBE ≈ 24.67%). Thus, we found evidence of both isolation by distance (IBD)^21^ and isolation by environment (IBE)^5^ in our population genetic dataset. IBD could be explained by geographical distance, extensive restriction and fragmentation of WTD forests^54^, physical barriers to gene flow (like the Yangtze River), or increasing fragmentation from other land uses in subtropical China (such as forest, agriculture and residential), all of which are commonly barriers to gene flow in plants^3^. Patterns of IBE often result from divergent selection between different environments; however, environmental factors can also shape gene flow through other processes (e.g. environmental differences affecting phenological differences among populations)^55^. Thus, IBE may not be due solely to selection but could also be explained by other diverse mechanisms associated with environmental factors^5^. Hence, multiple processes, associated with both geographic and environmental variables, all could have effectively disrupted gene flow between *E. henryi* populations, and broad-scale geographic and climatic factors appear to have shaped the spatial distribution of genetic variation in this species at both deeper evolutionary and more recent ecological time scales.

To examine the potential mechanism driving the pattern of IBE, we tested for loci under selection, in our AFLP dataset, and their associations with environmental/climatic variables. In identifying outlier AFLP loci, we sought to determine how selection might play a role in shaping genetic differentiation of the nine baps clusters of *E. henryi* along environmental clines. All four loci identified by both fdist and bayescan as undergoing putative diversifying selection (Fig. 5) were associated with environmental predictors across environmental gradients (Table S7), suggesting these regions of the genome are diverging and that climate may play a role. As expected, temperature and precipitation were estimated as the major driving factors influencing allele frequencies at outlier loci, consistent with other studies examining drivers of adaptive genetic divergence in plants^56^. These variables were also identified as important drivers of neutral genetic divergence by our SEM analysis (specifically bio5, maximum temperature of the warmest month, and bio15, precipitation seasonality). Altogether, these results are consistent with population genetic divergence under natural selection in divergent environments, suggesting this may have been the process generating IBE in this system.

In conclusion, by using phylogeographic, landscape genetic, and ecological niche modeling analysis together, we were able to identify the many factors, both historical and contemporary, that have shaped spatial genetic structure in this Tertiary relict species. At various spatial and evolutionary scales, we found that both geographical and environmental/climatic factors contribute to patterns of genetic structure. Geography played a predominant role at all levels – phylogeographic clades are broadly geographically structured, the deepest levels of divergence are associated with major geological or pre-Quaternary climatic events, and IBD primarily explained population genetic structure. However, environmental factors are clearly also important – climatic fluctuations since the LIG have likely contributed to phylogeographic structure, and the population genetic structure (in our AFLP dataset) was partly explained by IBE, which may have resulted from natural selection in environments with divergent climates. Thus, historical and contemporary geography and historical and contemporary environments have all shaped patterns of genetic structure in *E. henryi*, and, in fact, changes in the landscape through time have also been critical factors.

## Methods

### Plant material and sampling design

We obtained silica-dried leaf material from 38 populations of *Emmenopterys henryi* throughout its range (Table S1, Fig. 1A; see Supplementary Method S1 for more details of the study species). In each population, representative samples of 10–20 plants were taken, resulting in a total of 433 individuals. Total genomic DNA was extracted from the dried leaf tissue using a DNeasy plant tissue kit (Qiagen). All samples were sequenced at three intergenic spacer (IGS) regions of chloroplast DNA (cpDNA), while a subset of individuals (*n* = 212) was also sequenced at the entire internal transcribed spacer (ITS) region of nuclear ribosomal DNA (nrDNA). Of these 433 individuals, 394 (representing 37 populations; population S23 with 1 individual was excluded) were surveyed for AFLPs (Table S1; Fig. 1A). *Pinckneya bracteata* (Bartram) Raf., collected from the JC Raulston Arboretum (Raleigh, NC, USA), was selected as an outgroup for the phylogenetic analyses based on a previous molecular phylogenetic study of Rubiaceae^33^. Voucher specimens of this species and all sampled populations of *E. henryi* are stored at the Herbarium of Zhejiang University (HZU; Hangzhou, Zhejiang, China).

### DNA extraction, DNA sequencing and AFLP fingerprinting

For the phylogeographic DNA analyses, we sequenced three cpDNA-IGS regions (*psb*A–*trn*H, *trn*L–*trn*F, *trn*T–*trn*L) and the ITS region (i.e. ITS1 + 5.8S + ITS2). The primers and methodology for amplification of these four DNA regions were described in Shaw *et al*.^57^ and Gielly *et al*.^58^. Sequences were generated on an ABI 377XL DNA sequencer and were edited, assembled, and aligned in geneious v.4.8.5^59^. All sequences were deposited in GenBank (see Table S2 for accession numbers).

The AFLP protocol followed the procedure described by Vos *et al*.^60^, with minor modifications that included the use of fluorescent-dye-labeled primers (Applied Biosystems, Foster City, California, USA) for selective amplification in multiplex analysis. Selective primer pairs were initially screened on 50 individuals from 25 populations. Of the 64 primer pair combinations tested, nine pairs that gave the best results with respect to polymorphism and clarity of AFLP profiles (Table S3) were chosen for the full survey (further details in Supplementary Method S2). We also performed four separate test runs with 15 individuals and each chosen primer combination. These test runs confirmed that AFLP fragments were reproducible, resulting in an average error rate of 0.21% (±0.07) per fragment (see also Knowles & Richards^61^). The AFLP data matrix is available in Supplementary Dataset.

### Phylogeographical and population genetic data analyses

For both cpDNA and ITS, haplotype (*h*) and nucleotide (*π*) diversities were calculated for each population and the species overall using dnasp v.5.10^62^. Genealogical relationships of the haplotypes identified were inferred from a 95% statistical parsimony network constructed in tcs v.1.21^63^, with gaps (indels) coded as substitutions (A or T). For cpDNA, the permutation test implemented in permut was employed to compare parameters of population differentiation with unordered and ordered alleles (*G*_ST_ and *N*_ST_, respectively) based on 1000 random permutations^64^. Spatial analysis of molecular variance (SAMOVA), as implemented in samova v.1.0^65^, was used to identify regional groups of populations that are geographically homogeneous and maximally differentiated from each other. For cpDNA data, isolation by distance (IBD) effects^21^ were assessed by regressions of *F*_ST_/(1 − *F*_ST_) against the logarithm (log_10_) of geographic distance for all pairs of populations, or subsets thereof, following Rousset^66^.

For the AFLP dataset, we calculated the total number of AFLP fragments per population (*F*_T_), the percentage of fragments that are polymorphic within each population (*PPF*), Nei’s (1973) gene diversity (*H*_E_), and Shannon’s information index *I* following the method of Lynch & Milligan^67^ using aflpsurv v.1.0^68^. In addition, ‘frequency-down-weighted-marker values’ (*DW*; according to Schönswetter & Tribsch^69^), were calculated for each population. To infer the most likely number of population genetic clusters (*K*) in the AFLP dataset, we used three approaches. First, we used baps v.6.0^70^ to detect clusters of genetically similar populations and to estimate individual coefficients of ancestry (*q*) with regard to the detected clusters. Second, we utilized genalex v.6.4^71^ to run a principal coordinates analysis (PCoA), which non-hierarchically grouped the samples without prior knowledge of their source location. Third, we constructed an unrooted neighbour-joining (NJ) tree using the phylip package 3.6 with 1000 bootstraps (see Supplementary Method S3 for more details). To quantify variation in cpDNA sequences and AFLPs among populations and genetic clusters (as identified by SAMOVA), we performed analyses of molecular variance (AMOVAs) in arlequin v.3.5^72^ using *Φ*- statistics, respectively. The significance of fixation indices was tested using 10,000 permutations^73^.

### Divergence time estimation and demographic analyses based on cpDNA sequences

To relate differentiation among cpDNA haplotypes of *E. henryi* to pre-Quaternary and Quaternary events, we estimated divergence time under a Bayesian approach as implemented in beast v.1.8.0^74^. We estimated the divergence time using an unlinked substitution model (*psb*A–*trn*H: HKY; *trn*L–*trn*F: HKY; *trn*T–*trn*L: GTR+I) selected by jmodeltest v.2.0^75^. Given the intraspecific nature of our data, an uncorrelated lognormal distributed relaxed clock (ucld) model, and a coalescent model assuming constant population size were used to model the tree prior. Since no reliable fossils of *E. henryi* are currently known (see Introduction), we used the divergence time between *E. henryi* and *P. bracteata*, inferred from a fossil-calibrated cpDNA phylogeny of Rubiaceae, as a secondary calibration point to calibrate the stem node of *E. henryi*^33^ (node 1 in Fig. 1B: mean = 21.77 Ma; SD = 5.0 Ma; 95% CI = 7.92–33.64 Ma). For divergence time estimations, the Markov chain Monte Carlo (MCMC) was run for three independent 50 million generation chains, sampling from the chain every 5000 generations. The output was visualized using tracer v.1.5 (http://tree.bio.ed.ac.uk/software/tracer/) to ensure that parameter values were fluctuating at stable levels. Based on these results, the first 5000 trees were discarded as burn-in, and the remaining samples were summarized as a maximum clade credibility (MCC) tree with mean divergence times and 95% highest posterior density (HPD) intervals of age estimates in treeannotator v.1.8.0^76^. Finally, these results were summarized in a single tree visualized in figtree v.1.3.1 (http://tree.bio.ed.ac.uk/software/figtree/).

Tajima’s *D*^77^ and Fu’s *F*_S_^78^ neutrality tests were used to assess population demographic history. We also used arlequin to infer the demographic history of two main lineages identified (‘Northern’ vs. ’Southern’; see the Results Section) from their combined cpDNA-IGS sequences by mismatch distribution analysis (MDA) (further details in Supplementary Method S4).

### Present, past and future distribution modelling

Assuming *E. henryi* has not changed (and will not change) its climatic preference over at least the last glacial/interglacial cycle and into the future^79^, we reconstructed ENMs to determine the potential distribution of *E. henryi* across its range under present (1950*–*2000), past [the Last Glacial Maximum (LGM, *c*. 21 kya)^80^,^81^ and the Last Interglacial (LIG, *c*. 130–114 kya)^82^] and future (2080) climatic conditions using the maximum entropy method implemented in maxent v.3.2.1. In addition to the 38 distribution records included in this study, 76 herbarium-recorded presences were sourced from the Chinese Virtual Herbarium (www.cvh.org.cn) and the National Specimen Information Infrastructure of China (www.nsii.org.cn). Based on a total of 114 records, a current distribution model was developed using six bioclimatic data layers (annual mean temperature, annual precipitation, precipitation of wettest, driest, warmest and coldest quarter) available from the WorldClim database (www.worldclim.org)^83^ at 2.5 arc-min resolution for the present (1950–2000), assumed to be important for temperate species in East Asia^39^,^49^. This restricted dataset was used to avoid including highly correlated variables (data not shown), and thus to prevent potential over-fitting^84^. The established model was then projected onto the set of climatic variables simulated by the Model for Interdisciplinary Research on Climate (MIROC)^85^ to infer the extent of suitable habitat during the LGM and the LIG (see above). Future projections for 2080 were performed based on the Canadian Centre for Climate Modelling and Analysis model (CCCMA-CGCM31) under the A2 scenario that were provided by the CIAT downscaled GCM Data Portal (http://gisweb.ciat.cgiar.org/GCMPage/) with 30 arc-second resolution.

Model performance was evaluated using receiver operating characteristic (ROC) analyses in maxent. Values of the area under the ROC curve (AUC) between 0.7 and 0.9 indicate good fit^86^. We modelled the modern distribution 10 times, using different subsets of 70% of the localities to train the model and 30% to test the model, and visually compared AUC scores and jackknife tests of variable importance to assess consistency between runs.

### IBD and IBE analyses

To quantify the roles of geography and ecology in spatial genetic divergence, we quantified isolation by distance (IBD)^21^ and isolation by environment (IBE)^87^ using our AFLP dataset, because multi-locus datasets with highly variable markers are ideal for this purpose^5^,^88^,^89^. We computed population pairwise genetic distances (*F*_ST_) with arlequin using all putatively neutral (non-outlier) AFLP loci (i.e. 451 loci) described above. We obtained a total of 21 environmental and geographical variables for our study area, including 19 bioclimatic variables with 30 arc-second resolution (http://www.bioclim.org), a slope layer based on a digital elevation model with 1-km resolution from the USGS EROS database (http://eros.usgs.gov), and soil type from the Chinese soil taxonomy record^90^. We used resistance-based distances, to reflect biological connectivity between populations, instead of direct geographical distances^89^ (see Supplementary Method S5 for more details).

We then employed two related methods to quantify IBD and IBE: multiple matrix regression with randomization (MMRR)^88^ and structural equation modelling (SEM)^89^. Both methods use a series of regression-based analyses to quantify the effects of multiple explanatory variables on a single response variable, in this case genetic distance (*F*_ST_). MMRR provides a straightforward method for estimating linear regressions among distance matrices, while SEM allows for flexibility in defining the relationships among variables and utilizes latent variable modelling. For the MMRR analysis, we constructed an environmental distance matrix by taking the population pairwise Euclidian distances across all 21 environmental variables. We then analyzed the effects of environmental distance and geographic distance, as explanatory variables, on genetic distance, as the response variable, using the ‘MMRR’ function in R^88^ with 10,000 permutations. For the SEM analysis, we used all 21 environmental variables to construct an environmental distance latent variable, and then quantified the relationships between environmental distance, geographic distance, and genetic distance using the ‘lavaan’ package in R (http://cran.r-project.org/web/packages/lavaan/). Prior to analysis, all environmental, geographic, and genetic distance matrices were standardized. In each case, the analysis was used to test three alternative hypotheses: (1) only geographical distance contributes significantly to genetic distance, (2) only environmental distance contributes significantly to genetic distance, and (3) geographical distance and environmental distance each contribute significantly to genetic distance. We performed both analyses on AFLP pairwise genetic distances (*F*_ST_) for all populations.

### Potential AFLP loci under selection: detection of outlier loci and associations between allele frequencies and environmental variables

Two different basic approaches allow screening for AFLP loci that are putatively under selection: (i) a genome scan test procedure based on *F*_ST_ comparisons to identify the loci that are significantly different than expected under neutrality and a given demographic model; and (ii) correlative methods that look for associations between genetic variation and environmental variables. The key to using these approaches together is that they identify links between putative loci under selection and individual environmental variables. Hence, our primary purpose for using these tests was to identify the specific environmental variables that are potential drivers of genetic differentiation via divergent natural selection (e.g. IBE resulting from selection in divergent environments).

For the genome scan test, both the fdist^91^ and bayescan^92^ approaches were implemented on 457 polymorphic AFLP loci using the nine genetic clusters defined in the baps analysis (*K* = 9). Then, to detect associations between allele frequencies and environmental variables, we used Multiple Linear Regression (MLR)^93^ in R v.3.1.1 (R Development Core Team 2011) to identify potential adaptive loci that are under selection from current environmental factors. For outlier loci confirmed by both fdist and bayescan, we computed their population pairwise frequencies of AFLP alleles at the 37 sampling sites. We extracted values for the geographical coordinates of each population from 19 bioclimatic GIS data layers with 30 arc-second resolution (1950–2000; http://www.bioclim.org). We then regressed the allele frequencies of the retained outlier loci (dependent variables) on the selected environmental variables (explanatory variables; standardized) using the MLR model (see Table S4 for the retained allele frequencies and the selected environmental variables). Potential adaptive loci were identified as 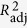 > 0.5 and significantly correlated to at least one explanatory variable^13^. Univariate regressions were then conducted for each variable individually to estimate its significance (see Supplementary Method S6 for more details).

## Additional Information

**How to cite this article**: Zhang, Y.-H. *et al*. Contributions of historical and contemporary geographic and environmental factors to phylogeographic structure in a Tertiary relict species, *Emmenopterys henryi* (Rubiaceae). *Sci. Rep*. **6**, 24041; doi: 10.1038/srep24041 (2016).

## Supplementary Material

Supplementary Information

Supplementary Dataset

## Figures and Tables

**Table 1:** Analyses of molecular variance (AMOVAs) based on cpDNA chlorotype frequencies, nrITS ribotype frequencies, and amplified fragment length polymorphism (AFLP) allele frequencies for populations of *E. henryi*.

Source of variation	cpDNA				nrITS			AFLPs		
	d.f.	Percentage of total variance (%)	Fixation indices	*G*_ST_/*N*_ST_	d.f.	Percentage of total variance (%)	Fixation indices	d.f.	Percentage of total variance (%)	Fixation indices
all										
Among populations	37	77.88	*Φ*_ST_ = 0.779	0.704/0.802**	37	55.78	*Φ*_ST_ = 0.558	36	34.41	*Φ*_ST_ = 0.344
Within populations	395	22.12			174	44.22		357	65.59	
South										
Among populations	22	82.27	*Φ*_ST_ = 0.823	0.757/0.868*	22	55.77	*Φ*_ST_ = 0.558	21	41.63	*Φ*_ST_ = 0.416
Within populations	229	17.73			103	44.23		212	58.37	
North										
Among populations	14	55.07	*Φ*_ST_ = 0.551	0.521/0.379	14	18.41	*Φ*_ST_ = 0.184	14	17.54	*Φ*_ST_ = 0.175
Within populations	166	44.93			71	81.59		145	82.46	
South + North										
Among regions	1	36.48	*Φ*_CT_ = 0.365		1	25.37	*Φ*_CT_ = 0.254	1	6.07	*Φ*_CT_ = 0.061
Among populations within regions	36	45.43	*Φ*_SC_ = 0.715		36	36.06	*Φ*_SC_ = 0.483	35	30.35	*Φ*_SC_ = 0.323
Within populations	395	18.1	*Φ*_ST_ = 0.819		174	38.56	*Φ*_ST_ = 0.614	357	63.58	*Φ*_ST_ = 0.364

For cpDNA, population differentiation for unordered (*G*_ST_) and ordered (*N*_ST_) haplotypes were calculated respectively at the different levels. d.f., degrees of freedom. *N*_ST_ significantly different from *G*_ST_ is shown in bold (**P < 0.01; *P < 0.05). Fixation index values were significant at all levels.

**Table 2:** Summary of mismatch distribution parameters and neutrality tests for four geographical groups of *E. henryi*.

Group	Parameter (τ)	Expansion time (*t*, Ma)	*SSD*	*P*	*H*_Rag_	*P*	Fu’s *F*_S_	*P*	Tajima’s *D*	*P*
Southeast population group	0.605 (0.000–2.184)	0.225 (0.000–0.811)	0.0003	0.723	0.189	0.638	−3.060	**0.039**	−1.204	0.110
Central-Southwest population group	3.569 (1.888–5.673)	NC	0.011	0.313	0.033	0.538	−0.696	0.469	−0.812	0.219
Northwest population group	0.511 (0.299–0.847)	0.190 (0.111–0.314)	0.002	0.186	0.152	0.446	−4.469	**0.005**	−1.474	**0.045**
Northeast population group	0.686 (0.359–1.011)	0.255 (0.133–0.376)	0.008	0.017	0.130	0.154	−3.388	**0.039**	−0.925	0.197

Estimates were obtained under models of spatial expansion using arlequin. NC, not calculated. There is a significant difference at the α = 0.05 level^85^ for Tajima’s *D* and Fu’s *F*_S_.

**Table 3:** Proportions of spatial genetic divergence explained by isolation by distance (IBD) and isolation by environment (IBE) based on SEM analysis and MMRR analysis using the neutral (non-outlier) AFLP dataset.

Model	IBD	IBE	TOTAL	COVAR.	CONTRIB. VARS.
SEM	0.360 ± 0.037***	0.181 ± 0.151***	0.541	0.133 ± 0.028	Temp.
MMRR	0.307***	0.247**	0.554	0.488*	Temp.

The sums of IBD and IBE (TOTAL), the covariation between these variables (COVAR.) and the primary contributors (CONTRIB. VARS.) to the environmental dissimilarity latent variable are listed. ***P < 0.001; **P < 0.005; *P < 0.05.
