# Supplementary material for: Contributions of historical and contemporary geographic and environmental factors to phylogeographic structure in a Tertiary relict species, Emmenopterys henryi (Rubiaceae)
Source: Sci Rep. 2016 May 3;6:24041. doi: 10.1038/srep24041 (PMC4853719; doi:10.1038/srep24041)
Supplement: Supplementary Information [file srep24041-s1.pdf]

Supplementary information for:

Title: Contributions of historical and contemporary geographic and environmental factors to phylogeographic structure in a Tertiary relict species, *Emmenopterys henryi* (Rubiaceae)

Authors: Yong-Hua Zhang[1], Ian J. Wang[2], Hans Peter Comes[3], Hua Peng, Ying-Xiong Qiu[1,*]

1. Key Laboratory of Conservation Biology for Endangered Wildlife of the Ministry of Education, and College of Life Sciences, Zhejiang University, Hangzhou 310058, China;

2. Department of Environmental Science, Policy, and Management, University of California, Berkeley, CA 94720, USA

3. Department of Ecology & Evolution, Salzburg University, A-5020 Salzburg, Austria

4. Laboratory of Biodiversity and Biogeography, Kunming Institute of Botany, Chinese Academy of Sciences, Kunming, Yunnan 650204, China

*Correspondence: Ying-Xiong Qiu. Fax: +86 571 86432273; E-mail: qyxhero@zju.edu.cn

<u>Supplementary information:</u>

<u>Figures S1–S3 ,Tables S1–S8 and Supplementary Methods S1–S6</u>

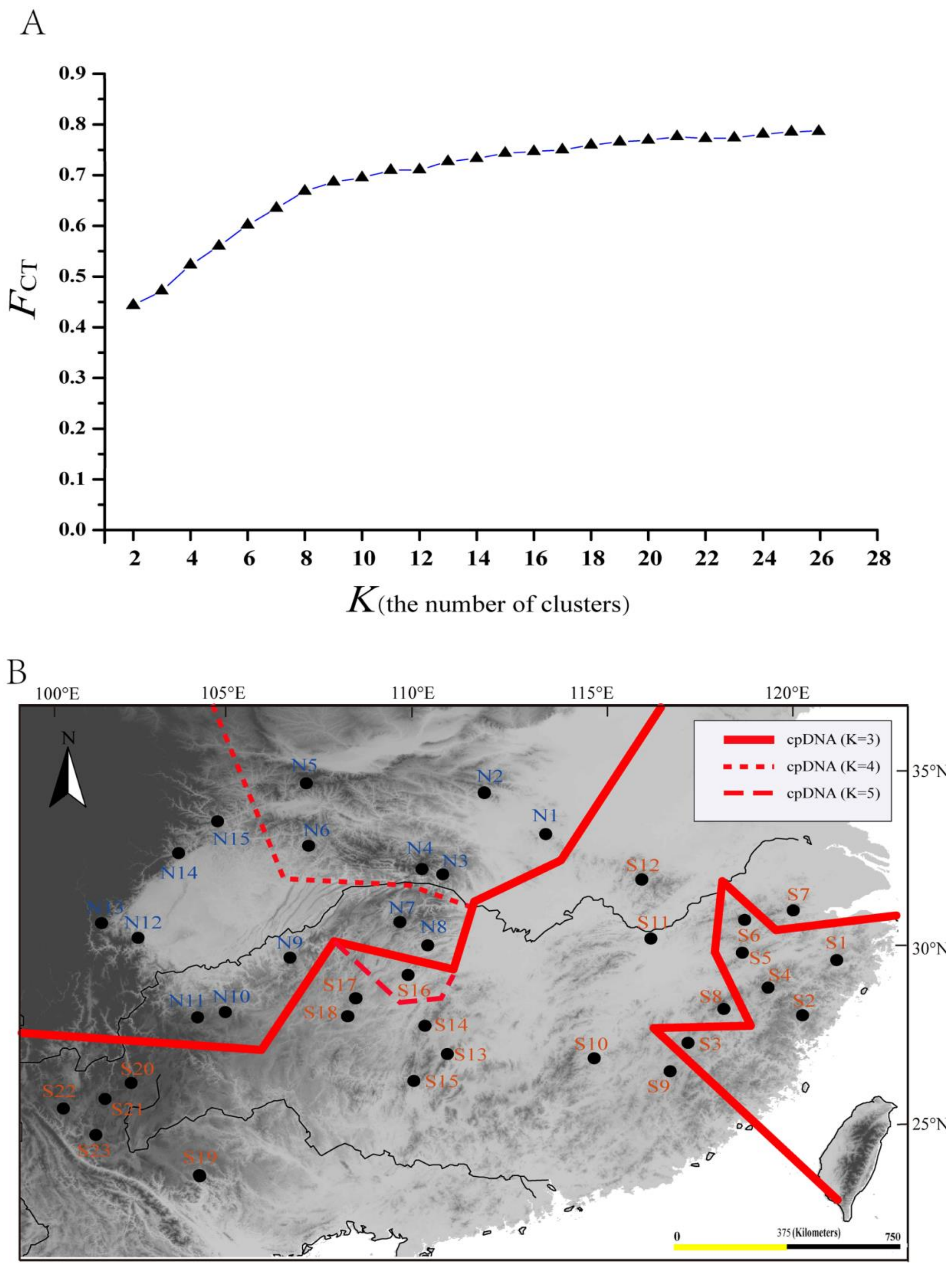

Figure S1. Results of the spatial analyses of molecular variance (SAMOVA) in 38 populations of *Emmenopterys henryi*. (**A**) FCT value obtained when searched for K = 2 to 26 groups using cpDNA data. (**B**) Results of clustering for K = 3 to 5 groups based on the cpDNA data. The map was drawn using ArcGIS v.9.3 (ESRI, Redlands, CA, USA), available at http://www.esri.com/software/arcgis/arcgis-for-desktop.

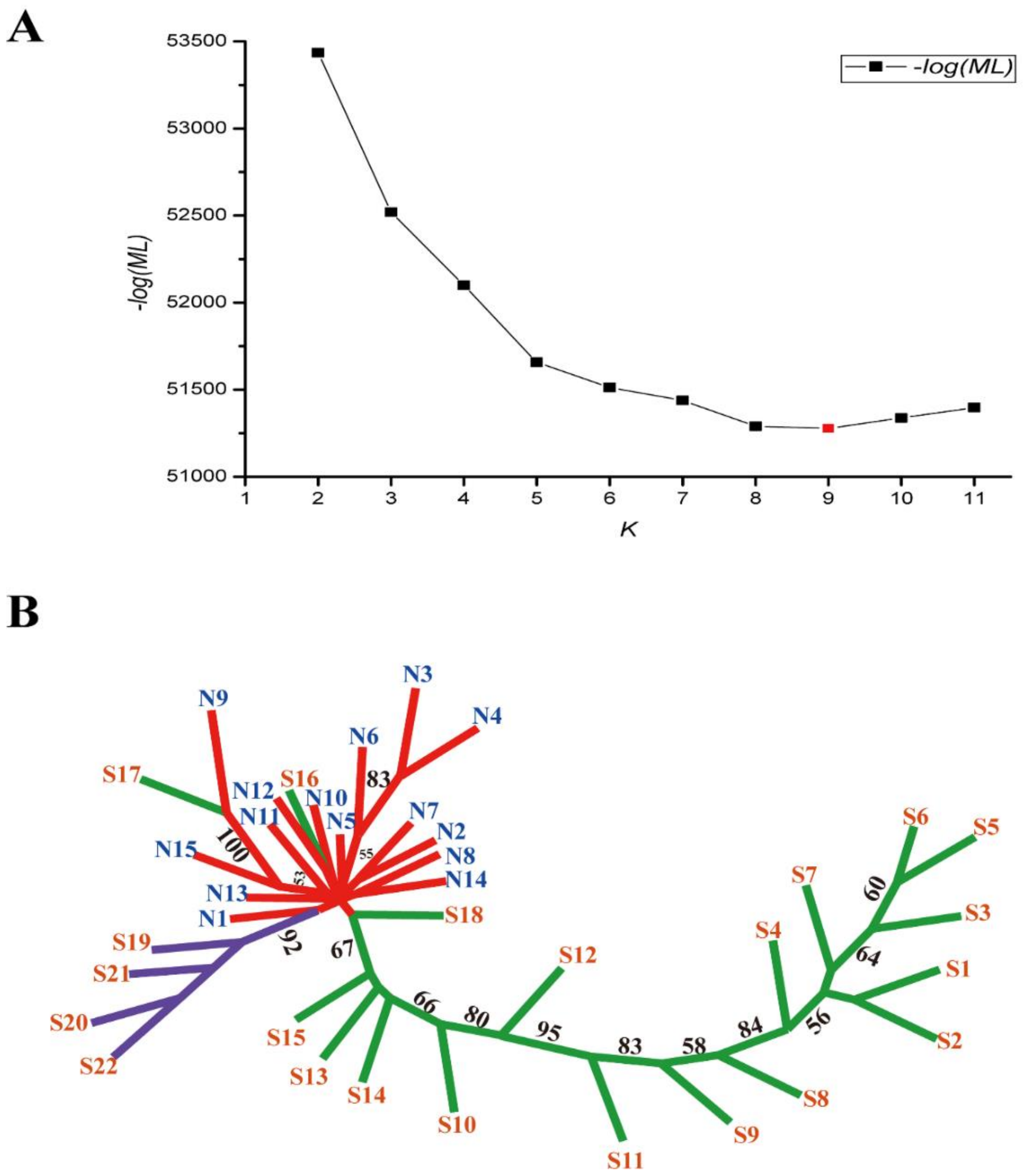

Figure S2. **(A)** Logarithm relationship between the number of groups (*K* – value) and the −log(lm) value for BAPS analysis of *E. henryi* for *K* = 2 to 11, and the best probability for clustering (*K* = 9). **(B)** Neighbor-joining network showing the genetic relationships among 37 populations of *E. henryi*, based on Nei ' s (1987) unbiased genetic distance (D)[1] calculated from 457 AFLP markers. Numbers around branches indicate bootstrap values from 1000 replicates. Population codes are identified in **Table S1**, also corresponding to the histogram **Fig. 3A**.

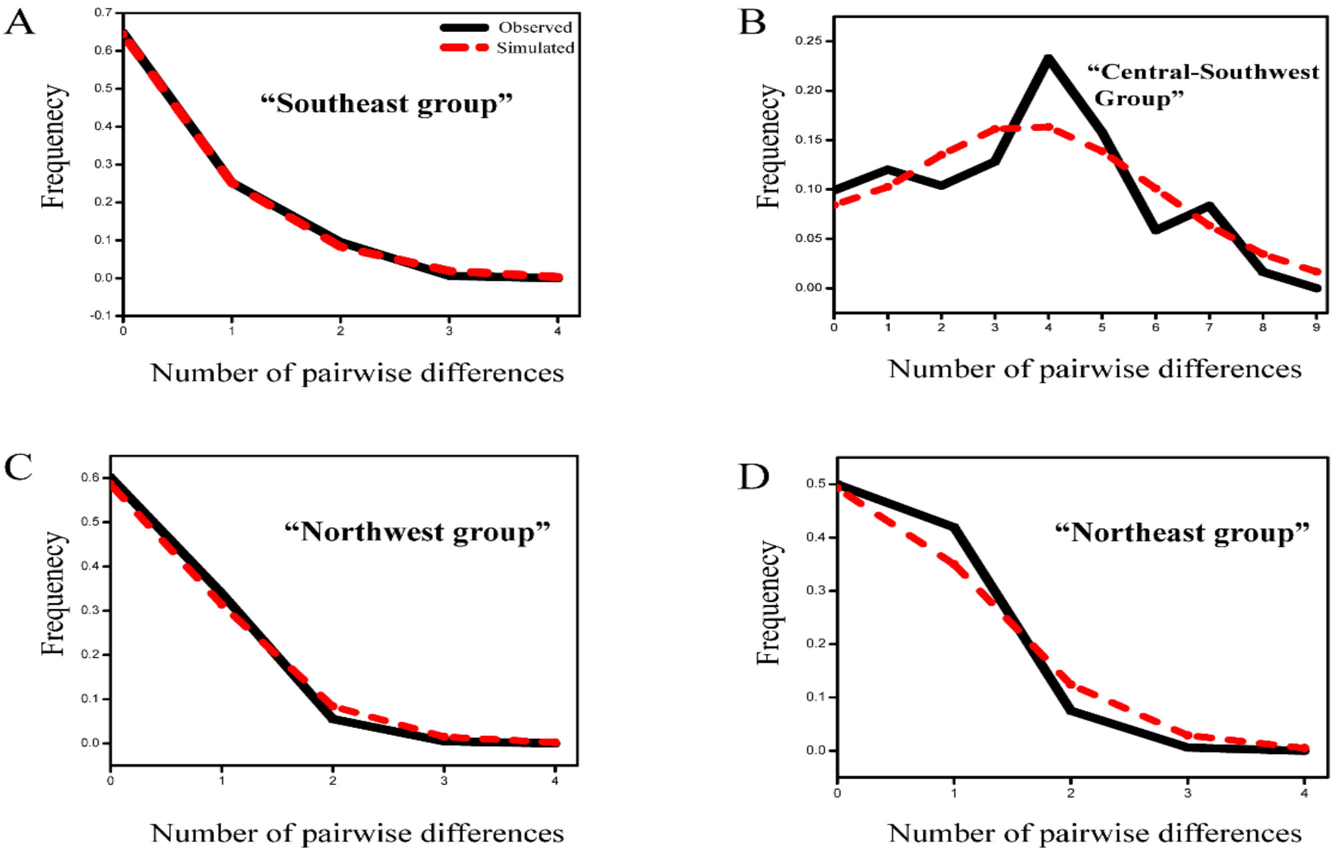

Figure S3. Frequency distributions of pairwise nucleotide differences for cpDNA sequence data of *E. henryi* in the group of **(A)** "Southeast group", **(B)** "Central-Southwest group", **(C)** "Northwest group" and **(D)** "Northeast group". The solid black line shows the observed distributions, whereas the dashed red line describes simulated data under a model of spatial (sudden stepwise) expansion.

Table S1 Geographic and genetic characteristics of 38 *Emmenopterys henryi* populations used in this study

					cpDNA			ITS		AFLPs				
population code	Locality	Latitude (N)/Longitude (E)	Altitude (m)	*n* (cpDNA/ITS/AFLP)	*h*	$\pi \times 10^{-3}$	Haplotypes (number of individuals)	*h*	$\pi \times 10^{-3}$	F_T	*PPF*	H_E	*I*	*DW*
Southern region														
S1	Mt.Dapan, Zhejiang	28°58'/120°32'	682−903	10/6/10	0.200	0.110	H2(9), H4(1)	0.600	1.560	312	68.27	0.099	0.147	17.265
S2	Wuyanling, Zhejiang	27°42'/119°39'	971−1091	12/6/11	0.561	0.350	H2(8), **H3(2)**, H4(1), **H5(1)**	0.733	2.690	298	65.21	0.095	0.140	17.104
S3	Jianning, Fujian	26°43'/116°51'	705−1040	11/6/9	0.509	0.280	**H8(4)**, H9(7)	0.533	2.090	299	65.43	0.089	0.131	10.363
S4	Mt.Jiulong, Zhejiang	28°22'/118°53'	663−1119	17/6/10	0	0	H2(17)	0.933	4.090	327	71.55	0.131	0.194	14.614
S5	Mt.Gutian, Zhejiang	29°15'/118°08'	439	13/6/11	0	0	H2(13)	0	0	254	55.58	0.027	0.039	12.029
S6	Mt.Huang, Anhui	30°09'/118°09'	1228−1298	13/6/11	0	0	H2(13)	0.600	1.560	308	67.40	0.104	0.154	12.620
S7	Mt.Tianmu, Zhejiang	30°21'/119°26'	1049−1033	11/6/11	0.327	1.080	H1(9), H2(2)	0.600	1.130	314	68.71	0.111	0.165	12.828
S8	Mt.Wuyi, Fujian	27°44'/117°38'	1247−1438	13/6/11	0.679	1.400	H1(7), H2(2), H6(3), **H7(1)**	0.333	0.870	317	69.37	0.151	0.224	11.901
S9	Ganjiangyuan, Jiangxi	25°58'/116°21'	802−836	11/5/9	0	0	H6(11)	0.600	3.130	300	65.65	0.113	0.166	9.302
S10	Mt.Jinggang, Jiangxi	26°35'/114°12'	791−813	11/6/10	0	0	H6(11)	0	0	315	68.93	0.098	0.147	13.823
S11	Mt.Lu, Jiangxi	29°32'/115°58'	871−927	12/6/10	0	0	H10(12)	0.533	2.090	306	66.96	0.122	0.179	12.240
S12	Tiantangzhai, Anhui	31°08'/115°45'	769−998	13/6/12	0	0	H1(13)	0.333	0.870	334	73.09	0.141	0.210	14.811
S13	Mt.Shunhuang, Hunan	26°25'/110°58'	1008-1084	10/6/12	0.600	1.060	H6(1), H19(6), **H22(3)**	0	0	325	71.12	0.124	0.186	18.181
S14	Heliping, Hunan	27°10'/110°19'	967-1017	13/5/12	0.513	0.280	H19(5), **H20(8)**	0	0	309	67.61	0.113	0.169	13.144
S15	Huaping, Guangxi	25°38'/109°54'	737	11/6/7	0	0	H21(11)	0	0	276	60.39	0.062	0.091	17.318
S16	Jishou, Hunan	28°20'/109°35'	300	8/6/11	0	0	H13(8)	0.333	0.430	298	65.21	0.110	0.162	13.106
S17	Yingjiang, Guizhou	27°56'/108°36'	1811	8/6/10	0.250	0.960	H12(1), H13(7)	0	0	333	72.87	0.121	0.183	11.631
S18	Shiqian, Guizhou	27°28'/108°29'	1146−1180	11/5/12	0.655	2.340	**H11(6)**, H12(3), H13(2)	0.600	0.780	351	76.81	0.153	0.231	15.991
S19	Wenshan, Yunnan	23°11'/104°47'	1647	11/6/12	0.691	1.120	H14(2), **H16(1), H17(6), H18(2)**	0	0	352	77.02	0.150	0.227	16.727
S20	Xundian, Yunnan	25°39'/103°04'	2063−2100	11/6/10	0	0	H14(11)	0	0	334	73.09	0.128	0.193	24.169
S21	Tuodan, Yunnan	25°14'/102°26'	1886	11/2/12	0.182	0.100	H14(1), **H15(10)**	0	0	266	58.21	0.068	0.103	19.742
S22	Zixishan, Yunnan	24°59'/101°25'	2206−2266	10/6/11	0	0	H14(10)	0	0	263	57.55	0.023	0.035	24.096
S23	Yuxi, Yunnan	24°16'/102°21'	1700	1/1/0	0	0	H14(1)	0	0	Null	Null	Null	Null	Null
	region mean				0.225	0.400		0.293	0.926	309	67.55	0.106	0.158	15.137
Northern region														
N1	Mt.Jigong, Henan	31°48'/114°05'	441−575	12/6/11	0	0	H28(12)	0	0	330	72.21	0.121	0.180	13.613
N2	Baotianman, Henan	33°29'/111°54'	750−996	17/6/12	0.404	1.600	**H27(3)**, H28(13), H29(1)	0.6	0.780	314	68.71	0.105	0.158	13.358
N3	Wudaoxia, Hubei	31°43'/110°13'	677	14/6/11	0.626	0.420	H28(8), H37(4), **H38(1), H39(1)**	0.533	1.390	347	75.93	0.134	0.204	13.303
N4	Shennongjia, Hubei	31°27'/110°24'	1024−1267	11/6/11	0.345	0.200	H28(9), H36(1), H37(1)	0.733	1.130	354	77.46	0.142	0.217	12.602
N5	Taibai, Shanxi	33°44'/107°25'	1119	20/6/11	0.479	2.370	H25(13), H26(7)	0.6	0.780	349	76.37	0.141	0.215	12.558
N6	Zhenping, Shanxi	32°02'/107°27'	1053	12/6/9	0.742	1.190	H25(2), H26(1), H28(4), H36(5)	0.6	0.780	345	75.49	0.145	0.219	10.312
N7	Mt.Badagong, Hunan	29°45'/110°03'	1263−1541	9/6/14	0.833	1.990	H12(1), H28(3), H31(3), **H32(1)**, H33(1)	0.6	0.780	314	68.71	0.145	0.221	15.913
N8	Zhangjiajie, Hunan	29°19'/110°25'	587−1074	11/6/11	0.618	0.480	H12(6), H31(4), **H35(1)**	0	0	342	74.84	0.143	0.216	11.268
N9	Mt.Jinfo, Chongqing	29°00'/107°04'	1127-1157	12/5/11	0	0	H12(12)	0.4	0.520	334	73.09	0.135	0.203	12.731
N10	Bijie, Guizhou	27°35'/105°25'	1160−1286	11/5/11	0	0	H12(11)	0	0	322	70.46	0.128	0.192	12.392
N11	Zhenxiong, Yunnan	27°28'/104°45'	1678	5/5/5	0	0	H12(5)	0	0	311	68.05	0.110	0.163	5.920
N12	Mt.Emei, Sichuan	29°35'/103°22'	813−1352	11/6/9	0.782	0.880	H12(4), **H23(3), H24(1), H40(3)**	0.733	1.130	339	74.18	0.162	0.243	12.891
N13	Xingou, Sichuan	29°54'/102°22'	1404−1424	11/6/12	0.327	0.180	H12(9), **H34(2)**	0.533	1.390	337	73.74	0.134	0.203	18.067
N14	Beichuan, Sichuan	31°49'/104°16'	528	11/5/9	0.182	0.100	H29(1), H30(10)	0.600	0.780	334	73.09	0.105	0.161	16.377
N15	Bifenggou, Gansu	32°41'/105°12'	893−965	14/6/13	0.538	2.150	H12(9), H26(1), H33(4)	0.533	0.700	362	79.21	0.147	0.223	40.100
	region mean				0.392	0.770		0.431	0.677	336	73.44	0.133	0.201	14.760
	species mean				0.291	0.540		0.347	0.828	320	69.93	0.117	0.176	14.984
	species total		300−2266	433/212/394	0.928	2.710		0.675	1.950	431	94.31	0.217	0.349	457.000

n, sample sizes for cpDNA/ITS/AFLPs analyses; *h*, haplotype diversity; *π*, nucleotide diversity; private haplotypes are noted by bold type; F_T, total number of fragments; *PPF*, percentage of polymorphic fragments; H_E, Nei's gene diversity[2]; *I*, Shannon's information index[3]; *DW*[4], the frequency down-weighed marker value, also called Rarity Index, calculated for populations.

Table S2 GenBank accession numbers of the cpDNA and ITS sequences of *Emmenopterys henryi* used in the this study

Taxon	Chlorotype	GenBank accession no. *psb*A-*trn*H	*trn*L-*trn*F	*trn*T-*trn*L	Ribotype	ITS
Pinckneya bracteata						
		KP347189	KP347230	KP347271		KP347148
Emmenopterys henryi						
	H1	KP347149	KP347190	KP347231	R1	KP347139
	H2	KP347150	KP347191	KP347232	R2	KP347140
	H3	KP347151	KP347192	KP347233	R3	KP347141
	H4	KP347152	KP347193	KP347234	R4	KP347142
	H5	KP347153	KP347194	KP347235	R5	KP347143
	H6	KP347154	KP347195	KP347236	R6	KP347144
	H7	KP347155	KP347196	KP347237	R7	KP347145
	H8	KP347156	KP347197	KP347238	R8	KP347146
	H9	KP347157	KP347198	KP347239	R9	KP347147
	H10	KP347158	KP347199	KP347240		
	H11	KP347159	KP347200	KP347241		
	H12	KP347160	KP347201	KP347242		
	H13	KP347161	KP347202	KP347243		
	H14	KP347162	KP347203	KP347244		
	H15	KP347163	KP347204	KP347245		
	H16	KP347164	KP347205	KP347246		
	H17	KP347165	KP347206	KP347247		
	H18	KP347166	KP347207	KP347248		
	H19	KP347167	KP347208	KP347249		
	H20	KP347168	KP347209	KP347250		
	H21	KP347169	KP347210	KP347251		
	H22	KP347170	KP347211	KP347252		
	H23	KP347171	KP347212	KP347253		
	H24	KP347172	KP347213	KP347254		
	H25	KP347173	KP347214	KP347255		
	H26	KP347174	KP347215	KP347256		
	H27	KP347175	KP347216	KP347257		
	H28	KP347176	KP347217	KP347258		
	H29	KP347177	KP347218	KP347259		
	H30	KP347178	KP347219	KP347260		
	H31	KP347179	KP347220	KP347261		
	H32	KP347180	KP347221	KP347262		
	H33	KP347181	KP347222	KP347263		
	H34	KP347182	KP347223	KP347264		
	H35	KP347183	KP347224	KP347265		
	H36	KP347184	KP347225	KP347266		
	H37	KP347185	KP347226	KP347267		
	H38	KP347186	KP347227	KP347268		
	H39	KP347187	KP347228	KP347269		
	H40	KP347188	KP347229	KP347270		

Table S3 Primer sequences and numbers of analysed loci for nine AFLP primer pairs of *E. henryi*

Primer pair ID	Primer EcoRI + 3 Selective PCR	Primer MseI + 3 Selective PCR	*n*	*DP*
eACAmCCA	5'-GAC TGC GTA CCA ATTC **ACA**-3	5'-GATGAGTCCTGAGTAA **CCA-**3'	66	96.97%
eAGGmCAA	5'-GAC TGC GTA CCA ATTC **AGG**-3	5'-GATGAGTCCTGAGTAA **CAA-**3'	45	88.89%
eAGTmCAG	5'-GAC TGC GTA CCA ATTC A**GT**-3	5'-GATGAGTCCTGAGTAA **CAG-**3'	38	94.74%
eACAmCGT	5'-GAC TGC GTA CCA ATTC **ACA**-3	5'-GATGAGTCCTGAGTAA **CGT**-3'	55	98.18%
eAGGmCCA	5'-GAC TGC GTA CCA ATTC **AGG**-3	5'-GATGAGTCCTGAGTAA **CCA-**3'	43	90.70%
eAGTmCCT	5'-GAC TGC GTA CCA ATTC A**GT**-3	5'-GATGAGTCCTGAGTAA **CCT**-3'	50	94.00%
eACAmCCT	5'-GAC TGC GTA CCA ATTC **ACA**-3	5'-GATGAGTCCTGAGTAA **CCT-**3'	69	98.55%
eAGGmCAT	5'-GAC TGC GTA CCA ATTC **AGG**-3	5'-GATGAGTCCTGAGTAA **CAT**-3'	49	89.80%
eAGTmCCC	5'-GAC TGC GTA CCA ATTC A**GT**-3	5'-GATGAGTCCTGAGTAA **CCC**-3'	42	92.86%

n, number of loci; *DP*, the degree of polymorphism

Table S4 Population code, the fragments frequencies of 6 outlier loci, the 8 selected environmental variables (standardized) for *E. henryi*

Population code	L128	L144	L294	L305	L354	L400	BIO2	BIO4	BIO8	BIO9	BIO10	BIO12	BIO15	BIO18
**S1**	0.007	0.946	0.946	0.007	0.567	0.378	0.711	-0.258	0.478	-0.089	-0.108	-1.212	1.833	-0.587
**S2**	0.005	0.952	0.952	0.005	0.716	0.489	-0.831	0.311	-0.597	-1.169	-0.781	0.301	-0.630	0.250
**S3**	0.006	0.950	0.950	0.006	0.950	0.006	0.882	0.060	0.791	-0.129	0.505	-1.612	1.517	-1.441
**S4**	0.009	0.925	0.925	0.009	0.925	0.563	-0.403	-0.426	0.523	-0.089	-0.475	-1.000	0.317	-0.788
**S5**	0.001	0.990	0.990	0.001	0.990	0.001	2.253	1.585	0.568	-1.969	0.444	-1.439	0.381	-1.584
**S6**	0.006	0.951	0.951	0.006	0.716	0.407	-0.574	0.596	1.239	0.191	0.505	0.963	-1.072	0.769
**S7**	0.006	0.945	0.945	0.006	0.945	0.407	-0.574	-0.485	1.508	1.031	1.179	0.247	1.960	2.461
**S8**	0.008	0.487	0.928	0.008	0.708	0.708	0.625	-0.528	-0.238	1.712	1.056	1.084	-0.314	0.284
**S9**	0.008	0.541	0.929	0.008	0.681	0.681	-0.831	0.547	-1.582	-0.049	0.137	1.540	-0.756	1.129
**S10**	0.113	0.007	0.946	0.007	0.946	0.946	-0.660	0.411	-1.537	-0.769	0.015	0.220	-0.946	-0.704
**S11**	0.007	0.700	0.700	0.007	0.941	0.700	0.025	0.016	1.105	1.392	1.240	0.548	-0.314	0.359
**S12**	0.008	0.363	0.431	0.008	0.931	0.721	-1.345	0.616	-0.865	-0.969	-1.027	1.414	-0.819	1.581
**S13**	0.007	0.007	0.509	0.007	0.939	0.939	-1.260	0.082	0.344	-0.129	0.505	-0.489	-0.314	-0.738
**S14**	0.006	0.006	0.938	0.006	0.938	0.938	-0.574	0.317	-0.955	0.591	0.750	0.837	-1.009	-0.194
**S15**	0.006	0.006	0.954	0.006	0.360	0.954	0.283	1.444	2.045	-0.969	1.608	-0.536	-0.440	-0.470
**S16**	0.006	0.006	0.006	0.006	0.946	0.006	-0.146	0.306	-0.865	0.791	0.934	1.316	-0.819	0.794
**S17**	0.009	0.009	0.302	0.302	0.929	0.172	0.025	-0.394	-0.955	0.991	0.260	1.365	-0.440	0.585
**S18**	0.010	0.010	0.301	0.143	0.429	0.597	0.025	0.690	1.239	0.071	1.791	-0.084	-0.693	-0.487
**S19**	0.009	0.009	0.009	0.430	0.009	0.920	-1.174	0.586	-2.029	-0.409	-0.291	1.254	-1.009	1.112
**S20**	0.173	0.061	0.115	0.462	0.235	0.924	-0.917	0.274	-1.671	0.031	-0.230	0.484	-1.009	-0.554
**S21**	0.977	0.002	0.002	0.977	0.002	0.977	-0.060	0.538	0.075	-1.369	-0.598	-0.672	-0.251	-0.914
**S22**	0.987	0.001	0.001	0.987	0.001	0.987	-0.317	0.324	0.344	-0.409	0.444	-0.385	-0.504	-0.830
**N1**	0.007	0.007	0.054	0.210	0.407	0.712	0.283	1.080	0.120	-1.769	-0.230	-1.459	0.696	-1.877
**N2**	0.006	0.006	0.006	0.006	0.946	0.243	2.339	-2.418	-0.194	1.712	-1.455	-1.091	1.644	-0.110
**N3**	0.009	0.009	0.009	0.056	0.406	0.486	-0.660	0.898	0.702	-0.689	-0.230	0.449	-1.072	0.292
**N4**	0.010	0.010	0.010	0.010	0.486	0.270	-0.232	0.997	0.702	-0.849	-0.230	0.479	-0.693	0.878
**N5**	0.010	0.010	0.404	0.056	0.334	0.485	-0.060	0.489	-0.373	-1.729	-1.210	-0.644	-0.188	-0.880
**N6**	0.012	0.012	0.012	0.069	0.537	0.672	-0.146	-2.191	0.075	1.672	-1.088	0.047	1.454	1.891
**N7**	0.007	0.007	0.121	0.081	0.738	0.738	-1.088	-0.287	-1.582	0.511	-0.414	1.831	-0.630	1.313
**N8**	0.009	0.009	0.334	0.056	0.706	0.582	-0.574	-0.212	-0.194	0.671	-0.230	1.397	-0.946	1.255
**N9**	0.009	0.009	0.211	0.211	0.925	0.104	2.339	-2.239	-0.910	0.871	-2.436	-1.086	1.644	-0.093
**N10**	0.008	0.008	0.055	0.008	0.155	0.334	0.625	-0.762	0.791	0.711	0.137	-1.066	1.391	-0.512
**N11**	0.017	0.017	0.122	0.122	0.900	0.243	-0.403	0.442	0.926	-0.009	1.240	-0.420	-0.504	-0.897
**N12**	0.012	0.012	0.130	0.012	0.432	0.265	-0.317	0.635	0.970	-0.129	1.424	0.158	-0.567	0.083
**N13**	0.008	0.050	0.008	0.244	0.930	0.720	0.025	0.360	0.836	-0.489	0.382	-0.590	0.633	-0.612
**N14**	0.008	0.008	0.193	0.008	0.683	0.193	0.111	-0.806	-0.283	-0.409	-1.578	-1.160	0.760	-0.822
**N15**	0.009	0.048	0.048	0.009	0.526	0.614	2.596	-2.596	-0.552	1.632	-1.945	-0.985	1.707	0.057

Table S5 Chloroplast DNA sequence polymorphism detected in *E. henryi* at three IGS-regions (*psb*A–*trn*H, *trn*L–*trn*F, *trn*T–*trn*L) and identifying 40 chlorotypes (H1−40)

Chlorotype	Nucleotide position																																											
	*psb*A-*trn*H						*trn*L-*trn*F														*trn*T-*trn*L																							
	1	2	7	71	136	198	2	84	127	149	203	207	263	290	344	351	418	529	609	723	5	39	154	197	213	231	232	242	252	281	285	289	292	296	302	331	353	376	414	436	570	578	707	727
H1	T	G	T	0	2^{b}	0	C	A	G	C	0	0	C	C	T	T	T	C	C	C	0	1	C	0	T	T	0	T	A	G	0	G	0	1	0	0	0	0	0	A	C	0	G	C
H2	.	.	G	0	2^{c}	0	.	.	.	A	0	0	.	A	.	.	G	.	.	.	0	1	.	0	-	.	0	.	.	.	0	.	0	1	0	0	0	0	0	.	.	0	.	.
H3	.	.	G	0	2^{c}	0	.	.	.	A	0	0	.	A	.	.	G	.	.	.	0	1	.	0	-	.	0	.	.	.	0	.	0	1	0	0	0	0	0	.	.	1^{u}	.	.
H4	.	.	G	0	2^{c}	0	.	.	.	A	0	0	.	A	.	.	.	.	.	.	0	1	.	0	-	.	0	.	.	.	0	.	0	1	0	0	0	0	0	.	.	0	.	.
H5	.	.	.	0	2^{c}	0	.	.	.	A	0	0	.	A	.	.	G	.	.	.	0	1	.	0	-	.	0	.	.	.	0	.	0	1	0	0	0	0	0	.	.	0	.	.
H6	.	.	.	0	2^{b}	0	.	.	.	A	0	0	.	A	.	.	.	.	.	.	0	1	.	0	-	.	0	.	.	.	0	.	0	1	0	0	0	0	0	.	.	0	.	.
H7	.	.	.	0	2^{b}	0	.	.	.	A	0	0	.	A	.	.	G	.	.	.	0	1	.	0	T	.	0	.	.	.	0	.	0	1	0	0	0	0	0	.	.	0	.	.
H8	.	.	G	0	2^{c}	0	.	-	A	A	0	0	.	A	.	.	G	.	.	.	0	1	.	0	-	.	0	.	.	.	0	.	0	1	0	0	0	0	0	.	.	0	.	.
H9	.	.	G	0	2^{c}	0	.	.	.	A	0	0	.	A	.	.	G	.	.	.	0	1	.	0	-	.	0	.	.	.	0	.	0	1	0	0	0	0	0	.	.	0	.	.
H10	.	.	.	0	2^{b}	0	.	.	.	A	0	0	.	A	.	.	.	.	.	.	1^{h}	1	.	0	-	.	0	.	.	.	0	.	0	1	0	0	0	0	0	.	.	0	.	.
H11	.	.	.	0	2^{b}	0	.	.	.	A	0	0	.	A	.	.	.	.	.	.	0	1	.	0	-	.	0	.	.	A	0	.	0	1	0	0	0	0	1^{t}	.	.	0	A	.
H12	G	T	.	0	2^{b}	0	.	.	.	A	0	0	.	A	.	.	.	.	.	.	0	1	.	0	-	.	0	.	.	.	0	.	0	1	0	0	0	0	0	.	T	0	.	.
H13	.	.	.	0	2^{b}	0	.	.	.	A	0	0	.	A	.	.	.	.	.	.	0	0^{j}	.	0	-	.	0	.	T	.	0	.	0	1	0	0	0	1^{s}	0	C	.	0	.	.
H14	.	.	.	0	2^{b}	0	.	.	.	A	0	0	.	A	.	.	.	.	.	T	0	1	.	0	-	.	0	.	.	.	0	.	0	1	0	0	0	0	0	.	.	0	.	.
H15	.	.	.	0	2^{b}	0	.	.	.	A	0	0	.	A	.	.	.	.	.	T	0	0^{j}	.	0	-	.	0	.	.	.	0	.	0	1	0	0	0	0	0	.	.	0	.	.
H16	.	.	.	0	2^{b}	0	.	.	.	A	0	0	.	A	.	.	.	.	.	T	0	1	.	1^{k}	-	.	0	.	.	.	0	.	0	1	0	0	0	0	0	.	.	0	.	.
H17	.	.	.	0	2^{b}	1^{e}	.	.	.	A	0	0	.	A	.	.	.	.	.	.	0	1	.	0	-	.	0	.	.	.	0	.	0	1	0	0	0	0	0	.	.	0	.	.
H18	.	.	.	0	2^{b}	1^{e}	.	.	.	A	0	0	G	A	.	.	.	.	.	.	0	1	.	0	-	.	0	.	.	.	0	.	1^{n}	0°	0	0	0	0	0	.	.	0	.	.
H19	.	.	.	0	2^{b}	0	.	.	.	A	0	1^{g}	.	A	.	.	.	.	.	.	0	1	.	0	-	.	0	.	.	.	0	.	0	1	0	0	0	0	0	.	.	0	.	.
H20	.	.	.	0	2^{b}	0	.	.	.	A	0	1^{g}	.	A	.	.	.	.	.	.	0	1	.	0	-	.	0	.	.	.	0	A	0	1	0	0	0	0	0	.	.	0	.	.
H21	.	.	.	0	2^{b}	0	T	.	.	A	0	0	.	A	.	.	.	.	.	.	0^{i}	1	.	0	-	.	0	G	.	.	1^{m}	.	0	1	0	0	0	0	0	.	.	0	.	.
H22	.	.	.	1^{a}	2^{b}	0	.	.	.	A	0	0	.	A	.	.	.	.	.	.	0^{i}	1	.	0	-	.	0	.	.	.	0	.	0	1	1^{p}	0	0	0	0	.	.	0	.	.
H23	G	T	.	0	2^{b}	0	.	.	.	A	0	0	.	A	.	.	.	.	.	.	0	1	.	0	-	.	0	.	.	.	0	.	1^{n}	1	0	0	0	0	0	.	T	0	.	.
H24	.	.	.	0	2^{b}	0	.	.	.	.	0	0	.	.	.	.	.	.	.	.	0	1	.	0	-	.	0	.	.	.	0	.	0	1	0	0	0	0	0	.	T	0	.	.
H25	.	T	.	0	2^{b}	0	.	.	.	A	0	0	.	A	.	G	.	.	.	.	0	0^{j}	.	0	-	.	1^{l}	.	.	.	0	.	0	1	0	0	0	0	0	.	T	0	.	.
H26	.	.	.	0	2^{b}	0	.	.	.	.	0	0	.	.	.	.	.	.	.	.	0	1	.	1^{k}	T	.	0	.	.	.	0	.	0	1	0	0	0	0	0	.	.	0	.	.
H27	.	T	.	0	2^{b}	0	.	.	.	A	1^{f}	0	.	A	.	.	.	.	A	.	0	1	T	0	-	G	0	.	.	.	0	.	0	1	0	0	1^{r}	0	0	.	.	0	.	.
H28	.	T	.	0	2^{b}	0	.	.	.	A	0	0	.	A	.	.	.	.	.	.	0	0^{j}	.	0	-	.	1^{l}	.	.	.	0	.	0	1	0	0	0	0	0	.	T	0	.	.
H29	.	T	.	0	2^{b}	0	.	.	.	A	0	0	.	A	.	.	.	.	.	.	0	1	T	0	-	G	0	.	.	.	0	.	0	1	0	0	1^{r}	0	0	.	.	0	.	.
H30	.	T	.	0	2^{b}	0	.	.	.	A	1^{f}	0	.	A	.	.	.	.	.	.	0	1	T	0	-	G	0	.	.	.	0	.	0	1	0	0	1^{r}	0	0	.	.	0	.	.
H31	G	T	.	0	2^{b}	0	.	.	.	A	0	0	.	A	.	.	.	T	.	.	0	1	.	0	-	.	0	.	.	.	0	.	0	1	0	0	0	0	0	.	T	0	.	.
H32	G	T	.	0	2^{b}	0	.	.	.	A	0	1^{g}	.	A	.	.	.	.	.	.	0	1	.	0	-	.	0	.	.	.	0	.	0	1	0	0	0	0	0	.	T	0	.	.
H33	.	T	.	0	2^{d}	0	.	.	.	A	1^{f}	0	.	A	.	.	.	.	.	.	0	1	T	0	-	G	0	.	.	.	0	.	0	1	0	0	1^{r}	0	0	.	.	0	.	.
H34	G	T	.	0	2^{b}	0	.	.	.	A	0	0	.	A	G	.	.	.	.	.	0	1	.	0	-	.	0	.	.	.	0	.	0	1	0	0	0	0	0	.	T	0	.	.
H35	G	T	.	0	2^{b}	0	.	.	.	A	0	0	.	A	.	.	.	.	.	.	0	0^{j}	.	0	-	.	1^{l}	.	.	.	0	.	0	1	0	0	0	0	0	.	T	0	.	.
H36	.	T	.	0	2^{b}	0	.	.	.	A	0	0	.	A	.	.	.	.	.	.	0	0^{j}	.	0	-	.	1^{l}	.	.	.	0	.	0	1	0	0	0	0	0	.	T	0	.	T
H37	.	T	.	0	2^{b}	0	.	.	.	A	0	0	.	A	.	.	.	.	.	.	0	0^{j}	.	0	-	.	1^{l}	.	.	.	0	.	0	1	0	1^{q}	0	0	0	.	T	0	.	.
H38	.	T	.	0	2^{b}	0	.	.	.	A	0	0	.	A	.	.	G	.	.	.	0	0^{j}	.	0	-	.	1^{l}	.	.	.	0	.	0	1	0	0	0	0	0	.	T	0	.	.
H39	.	T	.	0	2^{b}	0	.	.	.	A	0	0	.	A	.	.	G	.	.	.	0	0^{j}	.	0	-	.	1^{l}	.	.	.	0	.	0	1	0	1^{q}	0	0	0	.	T	0	.	.
H40	G	T	.	0	2^{b}	0	.	.	.	A	0	0	.	A	.	.	.	.	.	.	0	0^{j}	.	0	-	.	0	.	.	.	0	.	0	1	0	0	0	0	0	.	T	0	.	.

All sequences are compared to the reference haplotype H1. Numbers ‘0/1’ in sequences represent absence/presence of length polymorphisms. ‘2’ represents inversion sequence. A dash (–) denotes a single-nucleotide indel.

a TTTTTTTAAATGTCTATATTT

b TTTCCTTCTTGTTCTATTAAGAGGACG

c CGTCCTCTTAATAGAACAAGAAGGAAA

d TTTCCTTCTTGTTCTAGTAAGAGGACG

e ATAAGACTA

f TATGTATATCATATATAA

g ATATATAACACAA

h GTTAGCAACTATATATTTCTTA

i GTTAGCAACTATTATTTCTTA

j GATATGGATATAGAAAAGAATAAAAT

k AATTATCATTTAGTATTTAGA

l TATTAGATTAGATAGTAAAT

mTTTGAAATTCTTTTTTTACACTTCTATTATATATATTTATATATTTATTTTATATATTTTATTTATATTTATTTTTAT

n ATTCTTTTTTTACACTTCT

o CT

p TTACACTTCTATTATATATATTTATATATTTATATTATTTTACACTTCTATTATATATATTT

q GATATTTATATTATTATATTTATATATTTATATTATTT

r ATGATTCTATATCATAATCATAT

s GATGATTA

t GACATTCTACGCTTTCATTCATA

u GATTG

Table S6 ITS sequence polymorphism detected in *E. henryi* at ITS1 and ITS2 (the 5.8S excluded) regions and identifying 9 ribotypes (R1−9)

Ribotype	Nucleotide position									
	ITS1				ITS2					
	16	33	81	174	389	396	405	421	531	710
R1	C	G	G	G	G	G	C	C	C	C
R2	.	.	A	.	.	A	.	.	.	.
R3	.	.	.	.	.	A	.	.	.	.
R4	.	.	A	T	.	A	.	.	.	T
R5	T	A	A	.	.	A	.	.	T	.
R6	.	.	.	.	.	.	T	.	.	.
R7	.	.	.	.	.	.	.	T	.	.
R8	.	.	.	.	.	.	.	.	.	T
R9	.	.	.	.	T	.	.	T	.	.

All sequences are compared to the reference haplotype R1.

Table S7 Weightings for the makeup of the environmental distance latent variable in IBE analysis of *E. henryi*

Code	Variable	Weighting
BIO1	Annual Mean Temperature	0.321
**BIO4**	**Temperature Seasonality**	**1.042**
BIO5	Max Temperature of Warmest Month	0.456
**BIO7**	**Temperature Annual Range**	**0.847**
**BIO9**	**Mean Temperature of Driest Quarter**	**0.465**
BIO12	Annual Precipitation	-0.025
**BIO15**	**Precipitation Seasonality**	**0.465**
	slope	-0.046
	soil	0.043

Table S8 Results of outliers and environmental association analyses on AFLP loci of *E. henryi*

Outlier ID	Environmental variables (R^2_{adj})	Significant environmental variables
**L128**	**0.521**	BIO2,BIO4,BIO9,BIO10,BIO15
**L144**	**0.633**	BIO2,BIO12,BIO15,BIO18
**L294**	**0.802**	BIO2,BIO8,BIO12,BIO15,BIO18
**L305**	**0.703**	BIO2,BIO4,BIO9,BIO10,BIO12,BIO15
L354	0.408	BIO4,BIO10,BIO12,BIO15
L400	0.074	BIO4

Reference

1. Lynch, M. & Milligan, B. G. Analysis of population genetic structure with RAPD markers. *Mol. Ecol.* **3**, 91–99 (1994).
2. Nei, M. Analysis of gene diversity in subdivided populations. *P. Natl. Acad. Sci. USA* **70**, 3321–3323 (1973).
3. Shannon, C. E. & Weaver, W. The mathematical theory of information. *AT.&T. Tech. J.* **27**, 379–423 (1949).
4. Schönswetter, P. & Tribsch, A. Vicariance and dispersal in the alpine perennial *Bupleurum stellatum* L. (Apiaceae). *Taxon* **54**, 725–732 (2005).

Supplementary Method S1

Emmenopterys henryi is a tall canopy tree (20–30 m in height) with opposite simple leaves and terminal, corymbose to paniculiform cymes bearing numerous bisexual flowers with a 5-lobed calyx limb. In most inflorescences, however, some flowers have a 1-lobed calyx expanded into a white petaloid ‘calycophyll’, which is assumed to attract pollinators[1]. The fragrant flowers are pollinated by a wide variety of insects, including bees and butterflies[2]. The apically poricidal capsule contains numerous medium-sized seeds, which are broadly winged around and thus adapted for wind dispersal. Because of its small number of known populations (*c.* 114; Figs. 1A, 4A) and a low rate of natural regeneration, *E. henryi* is treated as ‘near threatened’ by the China Species Red List[3].

Supplementary Method S2

The AFLP protocol followed the procedure described by Vos *et al.*[4], with minor modifications that included the use of fluorescent-dye-labeled primers (Applied Biosystems, Foster City, California, USA) for selective amplification in multiplex analysis. Selective primer pairs were initially screened on 50 individuals from 25 populations. Of the 64 primer pair combinations tested, nine pairs that gave the best results with respect to polymorphism and clarity of AFLP profiles (Table S3) were chosen for the full survey. The *Eco*RI selective primers were labelled at the 5' end with WellRED *fluorescent* dyes (Beckman Coulter, Fullerton, CA). The sizes of the selective amplification products were determined using the ABI 3730xl DNA Analyzer (Applied Biosystems) with GeneScan 500 LIZ size standard (Applied Biosystems), performed by Tsingke Biotechnology Co., Ltd. (Beijing, China). We scored alleles by viewing the fluorescent peaks in the resulting chromatograms after calibration with the size standard, using GENEMARKER v.1.5 (SoftGenetics, State College, Pennsylvania, USA). AFLP bands were scored as present (1) or absent (0). Only bands between 60 bp and 360 bp that appeared above 1000 relative fluorescent units were scored to minimize scoring of false (artifact) bands. For each primer combination, negative amplification controls without template DNA resulted in no amplification products.

Supplementary Method S3

To infer the most likely number of population genetic clusters (*K*) in the AFLP dataset, we used three approaches. First, we used BAPS v.6.0[5] to detect clusters of genetically similar populations and to estimate individual coefficients of ancestry (*q*) with regard to the detected clusters. Despite the availability of some other software for Bayesian spatial analysis[6], we opted to use BAPS because of its efficiency in unveiling population structure[7] and its extensive usage for defining clusters of genetic variation using geographical information[8,9,10]. BAPS estimates the highest probability partition, i.e., the optimal number of clusters and assignments of the analyzed individuals. Both the frequencies of AFLP markers and the number of genetically divergent groups were treated as random variables. The analysis was repeated five times with the maximum number of clusters (*K*) set to 37 (corresponding to the total number of populations). Second, we utilized GENALEX v.6.4[11] to run a principal coordinates analysis (PCoA), which non-hierarchically grouped the samples without prior knowledge of their source location. Third, we constructed unrooted neighbour-joining (NJ) tree using the PHYLIP package 3.6 with 1000 bootstraps. we calculated Nei's (1987) unbiased genetic distance (*D*; according to Lynch & Milligan[12] among all possible pairs of populations from allele frequencies estimated in AFLPSURV using a Bayesian method with non-uniform prior distribution[13] under the assumption of Hardy-Weinberg equilibrium (HWE). One thousand distance matrices, calculated by bootstrapping over 457 AFLP fragments ('loci') were constructed using AFLPSURV. The procedures NEIGHBOR and CONSENSE of the program PHYLIP v.3.6[14] were then used to yield a neighbor-joining (NJ) network and to infer bootstrap confidence on tree branches.

Supplementary Method S4

For MDA, we tested the null hypothesis of a spatial expansion based on pairwise cpDNA haplotype differences. The goodness-of-fit was tested with the sum of squared deviations (*SSD*) between observed and expected mismatch distributions, and Harpending's (1994) raggedness index (H_{Rag})[15], using 10,000 parametric bootstrap replicates. If the selective neutrality tests (at least one) were significant and the null hypothesis was not rejected, the expansion parameter (τ) and its 95% confidence interval (CI) were translated to estimates of

absolute time (T, in number of generations), using the formula $T = \tau/2u$[16,17], where $u = \mu kg$; μ is the substitution rate per nucleotide site per year (6.225×10^{-10} s/s/y; inferred from the intraspecific BEAST analyses described earlier); k is the total cpDNA sequence length (here, 2163 bp; see the Results Section) used for analysis and g is the generation time in years (i.e. age of first reproduction, approximated as 6 years, as observed for *E. henryi* in cultivation)[18].

Supplementary Method S5

We obtained a total of 21 environmental and geographical variables for our study area, including 19 bioclimatic variables with 30 arc-second resolution (http://www.bioclim.org), a slope layer based on a digital elevation model with 1-km resolution from the USGS EROS database (http://eros.usgs.gov), and soil type from the Chinese soil taxonomy record[19]. For the 19 bioclimatic variables and the slope layer, we extracted values at every locality from each GIS data layer, using the 'raster' package in R (http://cran.r-project.org/web/packages/raster/), and calculated differences between localities to create a dissimilarity matrix for each variable. For the soil type, each sampling site was characterized by the soil type of its identified region[19]. The same type in two localities was assigned a distance of 0, partially-shared soil types were assigned a distance of 0.5, and fully different soil types were assigned a distance of 1, yielding a dissimilarity matrix for soil type.

We used resistance-based distances, to reflect biological connectivity between populations, instead of direct geographical distances[20]. The ENM for current climatic conditions generated previously resulted in a map of environmental suitability values ranging from 0 to 1 with higher scores reflecting more suitable habitat. We translated the suitability scores into resistance values[102] to generate a resistance layer for our study area. Resistance values were the reverse of the suitability scores (1 – suitability), because higher suitability is expected to have lower resistance[21]. We then compiled geographical distance matrices from the resulting resistance layers by calculating pairwise least-cost path distances between populations using the 'gdistance' package in R (http://CRAN.R-project.org/package=gdistance). Least-cost path distances are calculated by finding the minimum total cumulative cost (or resistance) between two points[21].

Supplementary Method S6

For the genome scan test, both the FDIST and BAYESCAN approaches were implemented on 457 polymorphic AFLP loci using the nine genetic clusters defined in the BAPS analysis (K = 9). First, we used the FDIST approach from Beaumont & Nichols[22], implemented in MCHEZA[23]. Loci with an unusually high F_{ST} are putatively under directional selection, while loci with low F_{ST} value are considered to be potentially under stabilizing selection. We simulated the neutral distribution of F_{ST} with 100,000 iterations at a significance level of 0.01. This method also implements a multi-test correction based on false discovery rates (FDR) that is fundamental for avoiding overestimation of the percentage of outliers (*e.g.* 1% of false positive with a threshold of 99%). To further minimize the risk of bias due to false positives, an additional analysis using BAYESCAN v.2.1[24] was conducted simultaneously, based on the binary AFLP data set. This method uses observations to update or newly infer the probability that a hypothesis may be true. To identify loci possibly under selection, the software provides Bayes factors and Posterior Odds (PO) scores. The latter, in particular, indicate how much more probable a model with selection is compared to a neutral model by calculating the ratio between their posterior probabilities[25]. After 10 pilot runs of 5000 iterations and an additional burn-in period of 50,000 iterations, we used 100,000 iterations (sample size = 5000, thinning interval = 20) to identify outlier loci. A posterior odds ratio greater than 10 (PO > 10)[24] was considered the criterion for determining that a marker is under directional selection. The false discovery rate was used to control for multiple tests.

Then, to detect associations between allele frequencies and environmental variables, we used Multiple Linear Regression (MLR)[26] in R v.3.1.1 (R Development Core Team 2011) to identify potential adaptive loci that are under selection from current environmental factors. We extracted values for the geographical coordinates of each population from 19 bioclimatic GIS data layers with 30 arc-second resolution (1950–2000; http://www.bioclim.org). We then implemented a principal component analysis (PCA) to reduce covariation in the dataset and obtain uncorrelated variables that account for most of the variation in the original data[26]. For outlier loci confirmed by both FDIST and BAYESCAN, we computed their population pairwise frequencies of AFLP alleles at the 37 sampling sites. We then regressed the allele frequencies of the retained outlier loci (dependent variables) on the selected environmental variables (explanatory variables; standardized) using the MLR model (see Table S4 for the retained

allele frequencies and the selected environmental variables). Potential adaptive loci were identified as $R^2_{adj} > 0.5$ and significantly correlated to at least one explanatory variable[27]. Univariate regressions were then conducted for each variable individually to estimate its significance.

References:

1. Chen, T. & Taylor, C. M. [Rubiaceae – *Emmenopterys*] *Flora of China* Vol.19 [Wu, Z. Y. & Raven, P. H. (eds)] [102] (Science Press; Missouri Botanical Garden Press, 1989–2013).
2. Cheng, X. M. *Study on the pollination biology of the state key protected species Emmenopterys Oliv.* (Henan Agricultural University, Henan, China, 2008).
3. Wang, S. & Xie, Y. *China species red list, vol. 1. Red List.* (Higher Education Press, Beijing, China (In Chinese) 2004).
4. Vos, P. *et al.* AFLP: a new technique for DNA fingerprinting. *Nucleic. Acids Res.* **23**, 4407–4414 (1995).
5. Corander, J., Marttinen, P., Siren, J. & Tang, J. Enhanced Bayesian modelling in BAPS software for learning genetic structures of populations. *BMC Bioinformatics* **9**, 539 (2008).
6. Guillot, G., Leblois, R., Coulon, A. & Frantz, A. C. Statistical methods in spatial genetics. *Mol. Ecol.* **18**, 4734–4756 (2009).
7. Corander, J. & Marttinen, P. Bayesian model learning based on predictive entropy. *J. Log. Lang. Inf.* **15**, 5–20 (2006).
8. Kropf, M., Comes, H. P. & Kadereit, J. W. Past, present and future of mountain species of the French Massif Central – the case of *Soldanella alpina* L. subsp *alpina*

(Primulaceae) and a review of other plant and animal studies. *J. Biogeogr.* **39**, 799–812 (2012).

9. Bitocchi, E. *et al.* Mesoamerican origin of the common bean (*Phaseolus vulgaris* L.) is revealed by sequence data. *P. Natl. Acad. Sci. USA* **109**, E788–E796 (2012).
10. Jones, F. A., Ceron–Souza, I., Hardesty, B. D. & Dick, C. W. Genetic evidence of Quaternary demographic changes in four rain forest tree species sampled across the Isthmus of Panama. *J. Biogeogr.* **40**, 720–731 (2013).
11. Peakall, R. & Smouse, P. E. GENALEX 6: genetic analysis in Excel. Population genetic software for teaching and research. *Mol. Ecol. Notes* **6**, 288–295 (2006).
12. Lynch, M. & Milligan, B. G. Analysis of population genetic structure with RAPD markers. *Mol. Ecol.* **3**, 91–99 (1994).
13. Zhivotovsky, L. A. Estimating population structure in diploids with multilocus dominant DNA markers. *Mol. Ecol.* **8**, 907–913 (1999).
14. Felsenstein, J. PHYLIP: phylogenetic inference program, v. 3.6 (University of Washington, Seattle, 2005).
15. Harpending, H. C. Signature of ancient population growth in a low resolution mitochondrial DNA mismatch distribution. *Hum. Biol.* **66**, 591–600 (1994).
16. Rogers, A. R. & Harpending, H. Population growth makes waves in the distribution of pairwise genetic differences. *Mol. Biol. Evol.* **9**, 552–569 (1992).
17. Rogers, A. R. Genetic evidence for a pleistocene population explosion. *Evolution* **49**, 608–615 (1995).
18. William, M. *Emmenopterys henryi*. *Pacific Horticulture,* **4**. (http://www.pacifichorticulture.org/articles/emmenopterys-henry/) (2005).

19. Gong, Z. *Chinese Soil Taxonomic Classification*. 5–215. (China Science Press, Beijing, China (in Chinese), 1999).

20. Wang, I. J., Glor, R. E. & Losos, J. B. Quantifying the roles of ecology and geography in spatial genetic divergence. *Ecol. Lett.* **16**, 175–182 (2013).

21. Wang, Y. H., Yang, K. C., Bridgman, C. L. & Lin, L. K. Habitat suitability modelling to correlate gene flow with landscape connectivity. *Landscape Ecol.* **23**, 989–1000 (2008).

22. Beaumont, M. A. & Nichols, R. A. Evaluating loci for use in the genetic analysis of population structure. *P. Roy. Soc. B.*: *Biol. Sci.* **263**, 1619–1626 (1996).

23. Antao, T. & Beaumont, M. A. Mcheza: a workbench to detect selection using dominant markers. *Bioinformatics* **27**, 1717–1718 (2011).

24. Foll, M. & Gaggiotti, O. A genome scan method to identify selected loci appropriate for both dominant and codominant markers: a Bayesian perspective. *Genetics* **180**, 977–993 (2008).

25. Fischer, M. C., Foll, M., Excoffier, L. & Heckel, G. Enhanced AFLP genome scans detect local adaptation in high-altitude populations of a small rodent (*Microtus arvalis*). *Mol. Ecol.* **20**, 1450–1462 (2011).

26. Zulliger, D., Schnyder, E. & Gugerli, F. Are adaptive loci transferable across genomes of related species? Outlier and environmental association analyses in Alpine Brassicaceae species. *Mol. Ecol.* **22**, 1626–1639 (2013).

27. Manel, S., Poncet, B. N., Legendre, P., Gugerli, F. & Holderegger, R. Common factors drive adaptive genetic variation at different spatial scales in *Arabis alpina*. *Mol. Ecol.* **19**, 3824–3835 (2010).
